# Tumour-Infiltrating Immune Cell-Based Subtyping and Signature Gene Analysis in Breast Cancer Based on Gene Expression Profiles

**DOI:** 10.7150/jca.37637

**Published:** 2020-01-14

**Authors:** Jingxin Jiang, Weiwei Pan, Yazhang Xu, Chao Ni, Dan Xue, Zhigang Chen, Wuzhen Chen, Jian Huang

**Affiliations:** 1 Department of Breast Surgery (Surgical Oncology), Second Affiliated Hospital, Zhejiang University School of Medicine, Hangzhou, China; 2 Department of Plastic Surgery, Second Affiliated Hospital, Zhejiang University School of Medicine, Hangzhou, China; 3 Key Laboratory of Tumor Microenvironment and Immune Therapy of Zhejiang Province, Hangzhou, China

**Keywords:** breast cancer, tumour-infiltrating immune cell, immunotyping, prognostic prediction model, prostaglandin D2 synthase (PTGDS)

## Abstract

Tumour-infiltrating immune cells have been indicated to play an important role in prognosis prediction and therapy sensitivity for breast cancer. In recent years, estimating the abundance of immune cells based on tumour transcriptome data has provided a novel way to analyse the clinical significance of various immune cell subsets. This study integrated breast cancer tissue transcriptome datasets from the Gene Expression Omnibus (GEO), the Cancer Genome Atlas-Breast Cancer (TCGA-BRCA) and the Molecular Taxonomy of Breast Cancer International Consortium (METABRIC) cohorts. A novel breast cancer immunotyping and a new prognostic model based on tumour-infiltrating immune cell subsets have been established, aiming to provide new clues regarding prognostic prediction and precision therapy for breast cancer. The key differentially expressed gene between different breast cancer immunotypes has also been identified. We performed unsupervised clustering analysis and construct a novel immunotyping which could classify breast cancer cases into immunotype A (B_cell^high^ NK^high^ CD8^+^_T^high^ CD4^+^_memory_T_activated^high^ γδT^low^ Mast_cell_activated^low^ Neutrophil^low^) and immunotype B (B_cell^low^ NK^low^ CD8^+^_T^low^ CD4^+^_memory_T_activated^low^ γδT^high^ Mast_cell_activated^high^ Neutrophil^high^) in luminal B, HER2-enriched and basal-like subtypes. The 5-year (85.7% *vs.* 73.4%) and 10-year OS (75.60% *vs.* 61.73%) of immunotype A population were significantly higher than those of immunotype B. A novel tumour-infiltrating immune cell-based prognostic model had also been established and the result immunorisk score (IRS) could serve as a new prognostic factor for luminal B, HER2-enriched and basal-like breast cancer. The higher IRS was, the worse prognosis was. We further screened the differentially expressed genes between immunotype A and B and identified a novel breast cancer immune-related gene, prostaglandin D2 synthase (PTGDS) and higher PTGDS mRNA expression level was positively correlated with earlier TNM stage. Immune-related signaling pathways analysis and immune cell subsets correlation analysis revealed that PTGDS expression was related with abundance of B cells, CD4^+^ T cells and CD8^+^ T cells, which was finally validated by immunohistochemical and immunofluorescence staining. We established a novel immunotyping and a tumour-infiltrating immune cell-based prognostic prediction model in luminal B, HER2-enriched and basal-like breast cancer by analyzing the prognostic significance of multiple immune cell subsets. A novel breast cancer immune signature gene PTDGS was discovered, which might serve as a protective prognostic factor and play an important role in breast cancer development and lymphocyte-related immune response.

## Introduction

Breast cancer ranks as the first in incidence rate among female malignant tumours and significantly impacts women's health 1, which is now considered a heterogeneous disease with different clinical and prognostic features 2. Although pathological molecular subtyping could classify breast cancer into four subtypes, luminal A, luminal B, human epidermal growth factor receptor 2 (HER2)-enriched and triple negative subtype, we could still find heterogeneity within each subtype, especially for luminal B and triple-negative breast cancer. Thus, it is necessary to explore new subtyping for prognostic prediction and indicators for efficacy evaluation to guide individualized treatment beyond the existing breast cancer molecular subtyping. With the development of tumour immunology, the interaction between tumour cells and tumour-infiltrating immune cells has gained widespread attention 3. Tumour-infiltrating immune cells, especially tumour-infiltrating lymphocytes (TILs), could play a key role as prognostic indicators in HER2-positive and triple-negative breast cancer (TNBC)^4^-^6^. Tumour-associated immune activation can improve clinical outcomes 7. Traditional studies have used flow cytometry, monoclonal antibody-based immunohistochemistry (IHC) or immunofluorescence (IF) detection to quantify the abundance and function of immune cell subsets 8. However, the identification of certain specific cell subset is still difficult, and it is hard to derive a landscape comprising all immune cell subsets 9. On the other hand, multiple gene expression signatures of primary breast cancer lesions have been used in clinical practice to predict patient outcomes. Three multigene expression assays (PCR-based OncotypeDX (Genomic Health Inc., Redwood City, CA, USA) 10, 11, microarray-based MammaPrint (Agendia Inc., Amsterdam, Netherlands)^12^, ^13^, NanoString-based PAM50 Prosigna Assay (NanoString Technologies Inc., Seattle, WA, USA)^14^, ^15^) have been used determine the risk of recurrence in patients with breast cancer. The genes included in these assays mainly played roles in cell proliferation, hormone receptors (HRs) and HER2 related signalling pathways 16. However, none of the current multigene expression assays demonstrate the relationship between primary tumours and the host immune system or contain prognostic-related immune genes to improve prediction accuracy.

With the rapid development of high-throughput genomic technologies in recent years, emerging bioinformatics tools have brought new opportunities for tumour immunological research. Different cell types have their specific gene expression profiles, which provides the possibility to estimate immune cell abundance. Researchers have begun to explore the landscape of infiltration immune cells from molecular level data such as gene chips or sequencing. A series of bioinformatic tools, such as MCPcounter 17, CIBERSORT 18 and deepTIL 19 have been developed to calculate the abundance and relative proportions of immune cell subsets in tumour tissue samples stably. Using the public transcriptome data with prognostic information, we could calculate the individual contents of tumour-infiltrating immune cells by CIBERSORT 20. Constructing a novel immune-related breast cancer typing and prognosis prediction model based on tumour infiltrating immune cells could be a currently available and promising method. Further studies on immune-related key regulatory genes and corresponding molecular mechanisms will help to improve the understanding of the tumour immune microenvironment.

This study utilized public data from databases such as the Gene Expression Omnibus (GEO), the Cancer Genome Atlas-Breast Cancer (TCGA-BRCA) and the Molecular Taxonomy of Breast Cancer International Consortium (METABRIC) to identify immune cell subsets associated with prognosis, perform clustering analysis for immunotyping and further established a least absolute shrinkage and selection operator (LASSO)-Cox prognostic model at immune cell level for breast cancer. We also screened the differentially expressed genes between different breast cancer immunotypes and identified a novel immune-related gene which was correlated with good breast cancer prognosis.

## Methods

### Data search strategy and collection

We conducted systematic searching in the GEO (https://www.ncbi.nlm.nih.gov/gds) database to identify breast cancer gene expression datasets with available clinicopathological and prognostic information. The search keywords were as follows: (“survival” OR “prognosis” OR “prognostic” OR “outcome” OR “death” OR “relapse” OR “recurrence”) AND (“breast cancer” OR “breast adenocarcinoma” OR “breast neoplasm” OR “breast tumour” OR “breast carcinoma”). Initially, 479 items were identified, but only 12 items met the inclusion and exclusion criteria at the same time. The inclusion criteria were as follows: (1) tissues from primary early-stage breast cancer in females; (2) gene mRNA expression profiling based on the GPL570 platform ([HG-U133_Plus_2] Affymetrix Human Genome U133 Plus 2.0 Array); (3) at least 50 samples from breast cancer cases; and (4) availability of information on overall survival (OS), recurrence-free survival (RFS), distant metastasis-free survival (DMFS), disease-free survival (DFS). The exclusion criteria included (1) duplicate cases from the same institute or hospital; (2) non-expression gene chips; (3) non-whole-genome chips; (4) breast cancer cases after neoadjuvant therapy; and (5) datasets with only breast cell lines included. TCGA-BRCA patient cohort data were downloaded from the TCGA website (https://cancergenome.nih.gov), and the METABRIC cohort data were downloaded from the cBioPortal website (https://www.cbioportal.org).

### Data pre-processing, normalization and integration

Raw data of all GEO datasets were extracted with the affy package in R software and individually normalized with the robust multi-array average (RMA) package, and batch effects were eliminated between experiments by applying the ComBat function in the SVA package. Microarray data were log2 transformed and normalized based on probe intensity values. Probe-symbol conversion and annotation were performed based on the GPL570 platform annotation database, hgu133plus2.db. Any probe that did not map to a gene ID was removed. Patient ID number, age at diagnosis, ER status, PR status, HER2 status, TNM stage, and histopathological grade were extracted from the clinical information provided, as well as the survival time and status including OS, DFS, RFS, and DMFS. The TCGA-BRCA and METABRIC datasets were processed independently.

### Estimation of immune cell abundance

CIBERSORT (http://cibersort.stanford.edu/) 21 was used with the three large datasets to calculate the absolute immunoscore and abundance of each immune cell subset by deconvolution method, which was well designed and had been validated with IHC in breast cancer. Both LM22 and LM7 gene signatures were used. LM22 was initially constructed to contain 547 genes and provide specific discrimination of 22 human immune cell phenotypes, including three B cell subsets, five CD4^+^ T cell subsets, CD8^+^ T cells, γδT cells, two natural killer (NK) cell subsets, three macrophage subsets, two dendritic cell (DC) subsets, monocytes, neutrophils and eosinophils. LM7 is established based on the CIBERSORT-LM22 that contains 375 genes and allows the estimation of abundance of 7 human immune cell types, including B cells, CD4^+^ T cells, CD8^+^ T cells, NK cells, γδT cells and MoMaDC (sum of macrophages, monocytes and DCs). LM7 could provide more precise estimation for the abundance of γδT cells 19. Thus, the abundance of γδT cells estimated using LM7 is considered eligible for further analysis, while the abundance of other immune cell subsets was estimated using LM22. CIBERSORT derives a *P* value for the deconvolution of each sample using Monte Carlo sampling, providing measurement confidence for each estimation. Samples with *P* < 0⋅05 were considered accurate and could be included for further analysis.

### Histological validation and clinical data collection

We collected formalin-fixed paraffin-embedded sections from 98 breast cancer patients who underwent surgical treatment at the Second Affiliated Hospital of Zhejiang University School of Medicine from August 2014 to August 2017. The related basic clinicopathological and survival information was also collected after receipt of informed consent and approval from the ethics committee. Gene expression and co-localization were validated by monoclonal antibody-based immunohistochemistry and immunofluorescence. Immunohistochemical staining by Envision method was performed on formalin-fixed paraffin-embedded slides, which had been dewaxed and rehydrated before antigen retrieval step. The intensity and frequency were used as evaluation indexes based on the brown staining of PTGDS. The intensity was divided into: negative (0), weak positive (1), positive (2), strong positive (3). The frequency was divided into: 0% ~ 10% (1), 11% ~ 30% (2), 31% ~ 50% (3), 51% ~ 75% (4), 76% ~ 100% (5). Comprehensive score = intensity*frequency. For immunofluorescence staining, formalin-fixed paraffin-embedded slides were heat-repaired by citrate buffer for 2 minutes, incubated with primary antibody at 4℃ overnight, incubated with fluorescein-labelled secondary antibody at room temperature, stained with DAPI and photographed by laser confocal microscopy.

### Bioinformatical and statistical analysis

All statistical analyses were conducted using R studio software (Version 1.1.414; http://www.rstudio.com/products/rstudio). This study was conducted and reported in accordance with the TRIPOD guidelines. The molecular subtyping of breast cancer in patients were all determined with a PAM50 identifier function provided by the genefu package. Unsupervised hierarchical clustering analysis was conducted within breast cancer samples and cell subsets with the hclust function. Unsupervised hierarchical clustering analysis could discriminate breast cancer samples based on different immunotypes. Survival analysis was performed by the survival and survminer packages. Survival curves were constructed by the Kaplan-Meier method and compared by the log-rank test. Hazard ratios (HRs) were calculated using both univariable and multivariable Cox proportional hazards regression models. The LASSO-Cox regression model with LASSO penalty was used to select the most specific prognostic cell subpopulations among the 22 immune cell subsets, and the optimal values of the penalty parameter λ were determined by tenfold cross-validations. A new prognostic variable, immunorisk score, was then established based on the abundance of the selected immune cells using Cox regression coefficients in the integrated GEO dataset, which was further validated in the TCGA-BRCA and METABRIC cohorts. A multivariable Cox regression model was used to determine independent prognostic factors. Group comparisons were performed for continuous and categorical variables using one-way ANOVA and the χ test, respectively. Correlations among cell subsets were analysed by Pearson's correlation test. All statistical tests were two-sided, and *P* < 0⋅05 was considered statistically significant.

## Results

### Overview of included breast cancer cohorts

**Figure 1:**
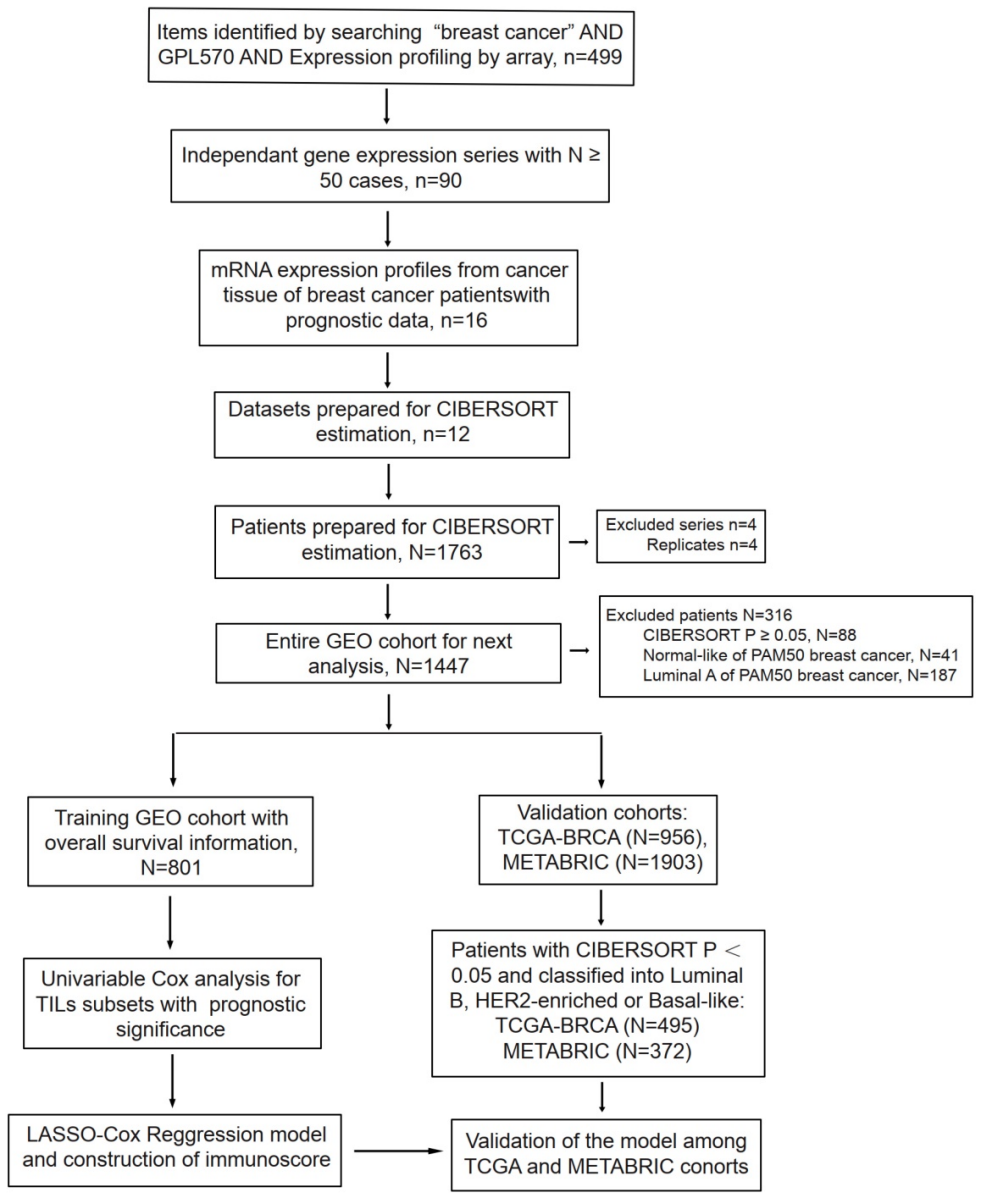
Flowchart of data collection and analysis. LASSO, least absolute shrinkage and selection operator.

**Figure 2:**
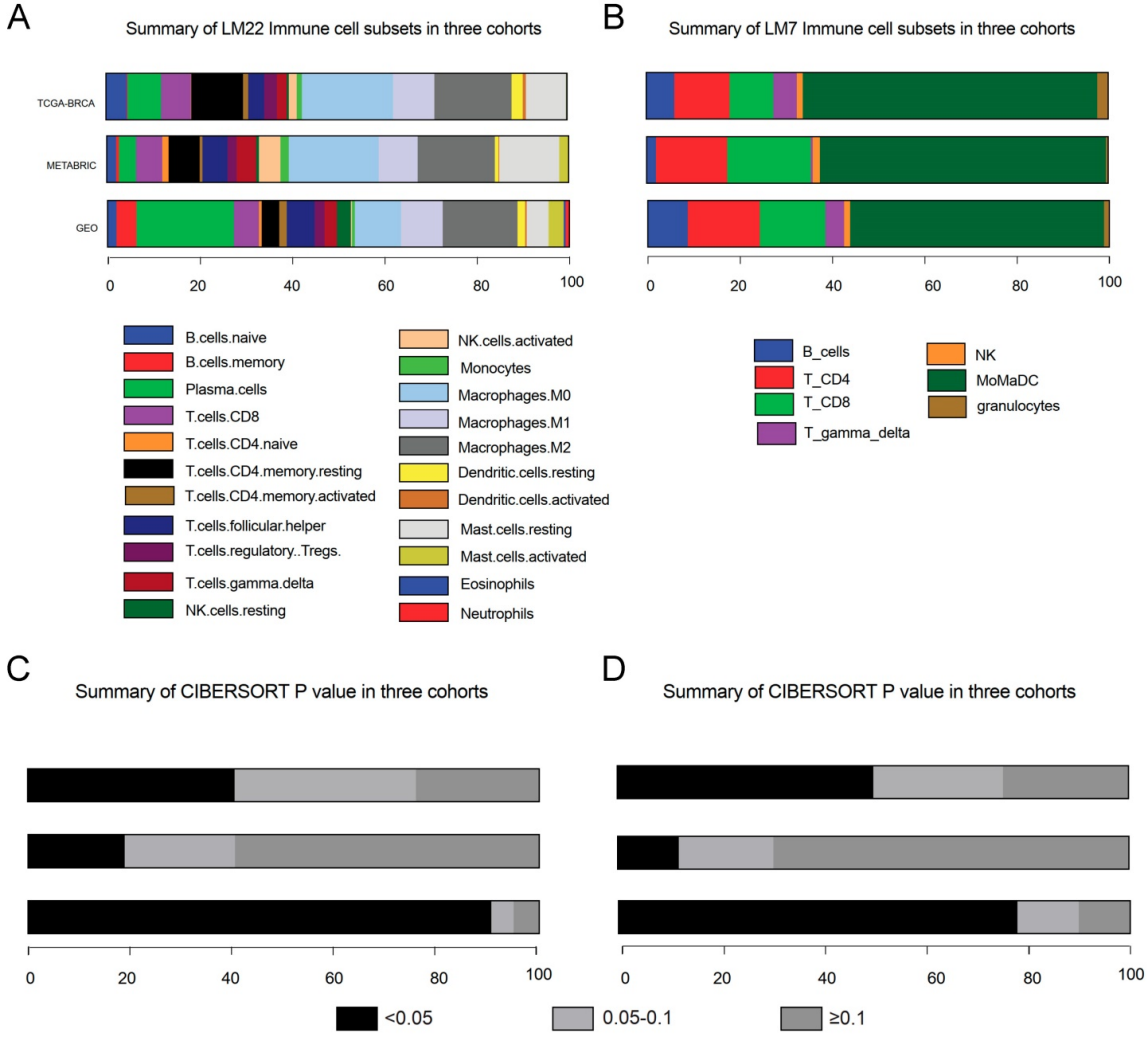
Abundance of immune cells subsets and P value estimated based on CIBERSORT-LM22 (A & C) and CIBERSORT-LM7 (B & D). MoMaDC, macrophages, monocytes and DCs.

After data incorporation and filtration, 801 breast cancer samples and 964 normal tissue samples from 12 GEO datasets with prognostic information were included for further analysis, with a mean follow-up time of 5.54 years (Figure 1 & Table S1). The clinicopathologic characteristics of breast cancer patients form the GEO cohort, TCGA cohort and METABRIC cohort were listed in Table 1. The estimated abundance of each immune cell subset was calculated by deconvolution method based on CIBERSORT-LM22 and CIBERSORT-LM7 in the TCGA-BRCA, METABRIC and GEO cohorts and was shown in Figure 2. The CIBERSORT* P* value < 0.05 indicates precise estimated result.

### Abundance and distribution of tumour-infiltrating immune cells

Firstly, we compared the estimated abundance and distribution of tumour-infiltrating immune (TILs) subsets in different breast cancer subtypes. TILs were more abundant in HER2-enriched and basal-like breast cancer types (Figure S1). In detail, we observed more B cells and M0/M1 macrophages in HER2-enriched and basal-like subtypes than in luminal A and B subtypes, but there were fewer CD8^+^ T cells, mast cells and M2 macrophages in HER2-enriched and basal-like subtypes than in luminal A and B subtypes. We found that the absolute immunoscore reflected the abundance of total tumour-infiltrating immune cells and was positively correlated with poor pathological characteristics, such as HR negativity (*P* < 0.001), lymph node positivity (*P* = 0.01) and higher histological grade (*P* < 0.001) (Figure S2). Further analysis showed that a higher percentage of CD8^+^ T cells and plasma cells were present in the lymph node-positive tumours, whereas a higher percentage of activated mast cells, Treg cells, resting NKs and DCs were present in the lymph node-negative tumours. With histological grade increasing, the percentage of macrophages, naive B cells and neutrophils rose, while γδT cells, Treg cells and mast cells decreased (Figure S2).

To screen for prognostic-associated immune cell subsets, we performed univariate Cox survival analysis and found there was a significant correlation between immune cell abundance and survival rate in luminal B, HER2-enriched and basal-like breast cancer (Figure S3). Further subgroup analysis suggested that, all tumour-infiltrating immune cells were grouped into 3 subsets: survival-favourable immune cell subsets including B cells, CD8^+^ T cells, activated CD4^+^ memory T cells, M1 macrophages and NK cells; survival-unfavourable immune cell subsets including Treg cells, M0/M2 macrophages, activated mast cells, neutrophils and γδT cells; and neutral immune cell subsets including DCs, monocytes, eosinophils and T follicular helper (Tfh) cells (Table 2). Pearson's correlation coefficients between immune cell subsets with clinical significance in the GEO cohort are shown in Figure S4.

### Establishment of a tumour-infiltrating immune cell-based prognostic model

Based on the above results, we used LASSO-Cox regression to screen variables and build a tumour-infiltrating immune cells-based prognostic model for luminal B, HER2-enriched and basal-like subtypes using data from the GEO cohort. Among the 22 immune cell subsets with clinical significance, 7 key immune cell subsets were included in the tumour-infiltrating immune cell-based prognostic model. A risk score called the immunorisk score (IRS) was calculated based on the abundance of tumour-infiltrating immune cells.

Immunorisk score = 2^((-0.056)*B cell+(-0.017)*CD8^+^ T cell+0.151*γδT+(-0.060)*NK cell+(-0.165)* activated CD4^+^ memory T cell+(0.099)*activated mast cell+(0.177)*neutrophils)

**Figure 3:**
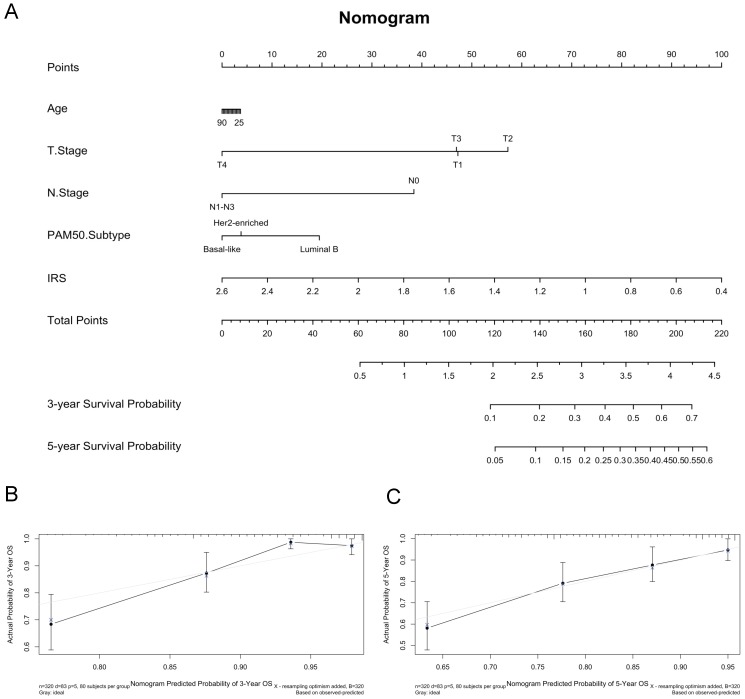
Construction of predictive nomogram based on age, T stage, N stage, pam50 subtypes and immunorisk score in GEO cohort. (A) Predictive nomogram. (B-C) Calibration curve of the nomogram with 3-year overall survival and 5-year overall survival. IRS, immunorisk score.

**Figure 4:**
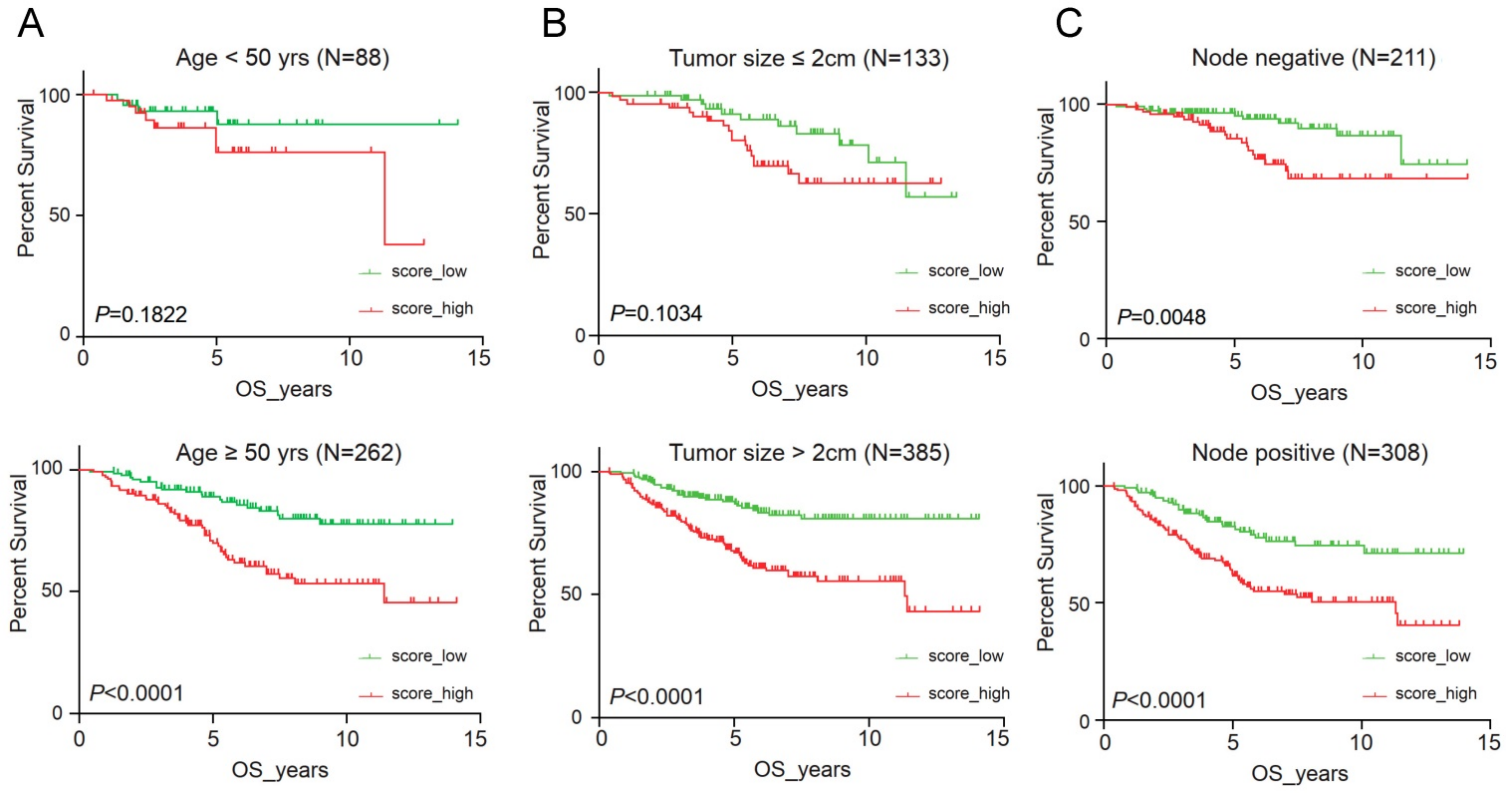
Clinical significance of immunorisk score grouped by clinicopathological features as age (A), tumour size (B) and lymph node (C) in GEO cohort. Score_high, immunorisk score high; Score_low, immunorisk score low.

It suggested that higher IRS had worse OS (*P* < 0.001), DFS (*P* < 0.001), RFS (*P* = 0.04) and DMFS (*P* < 0.001) using quartile cut-off values, indicating that IRS could serve as a novel prognostic marker (Figure S5). Nomogram predicting 3-year and 5-year OS was also constructed, with a C-index of 0.71 (95%CI: 0.64-0.78), suggesting this immune cell-based model could well reflect the prognosis (Figure 3 & Table S2). Subgroup analysis indicated that IRS was more accurate in high-risk groups, such as patients with age greater than 50 years old, tumours larger than 2 cm, or positive lymph nodes (Figure 4).

**Figure 5:**
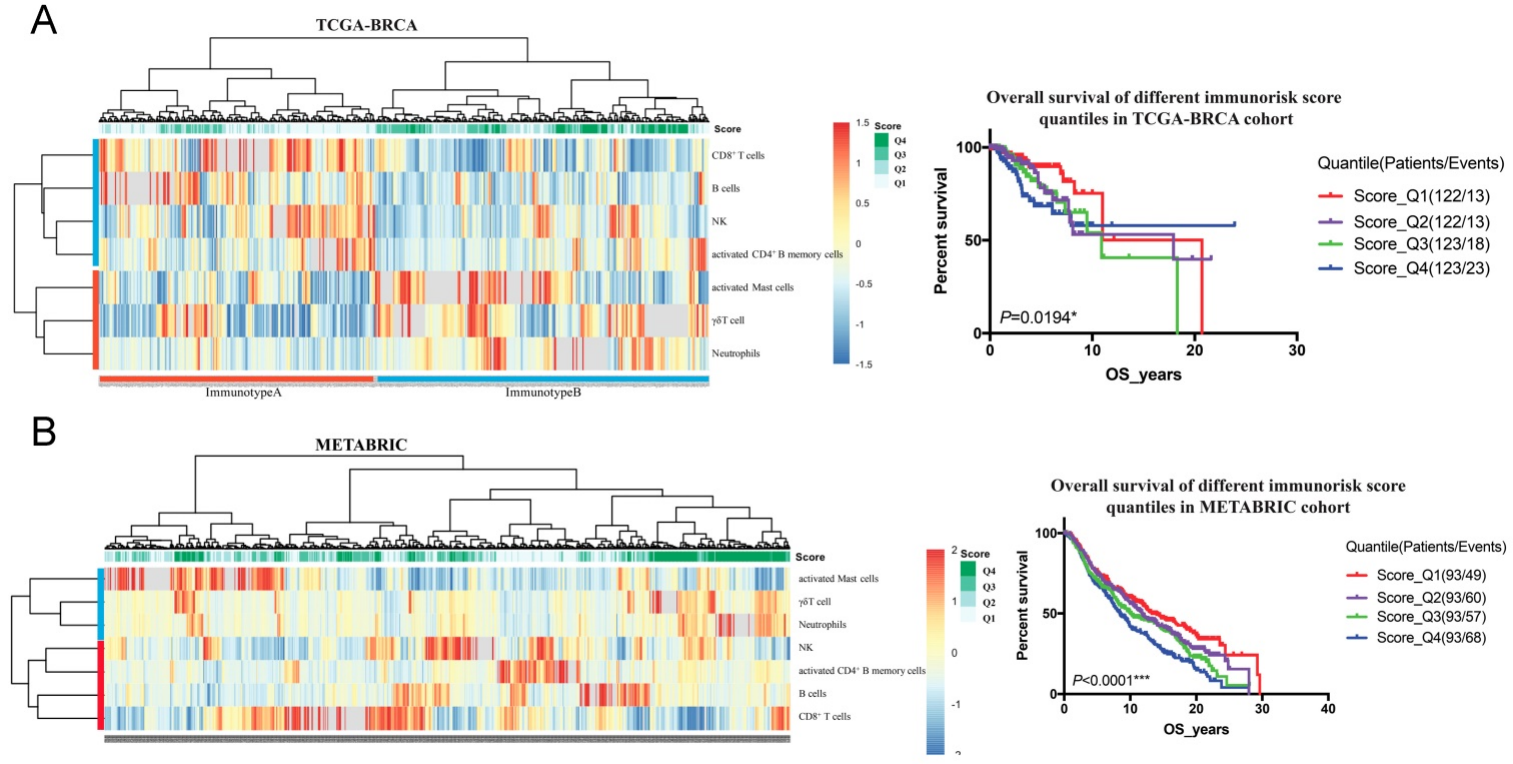
Clinical significance validation of immunorisk score in TCGA-BRCA (A) and METABRIC (B) cohorts. (P<0.05)

To validate the prognostic multivariable Cox regression model, we further perform the regression model in TCGA-BRCA and METABRIC cohorts. Higher IRS was a statistically significant factor associated with poor OS in both the TCGA-BRCA cohort (HR 11.80, 95% CI: 3.86-36.13, *P* < 0.001) and the METABRIC cohort (HR 1.24, 95% CI: 1.02-1.51, *P* = 0.035) (Figure 5).

### Clustering analysis for breast cancer immunotyping

**Figure 6:**
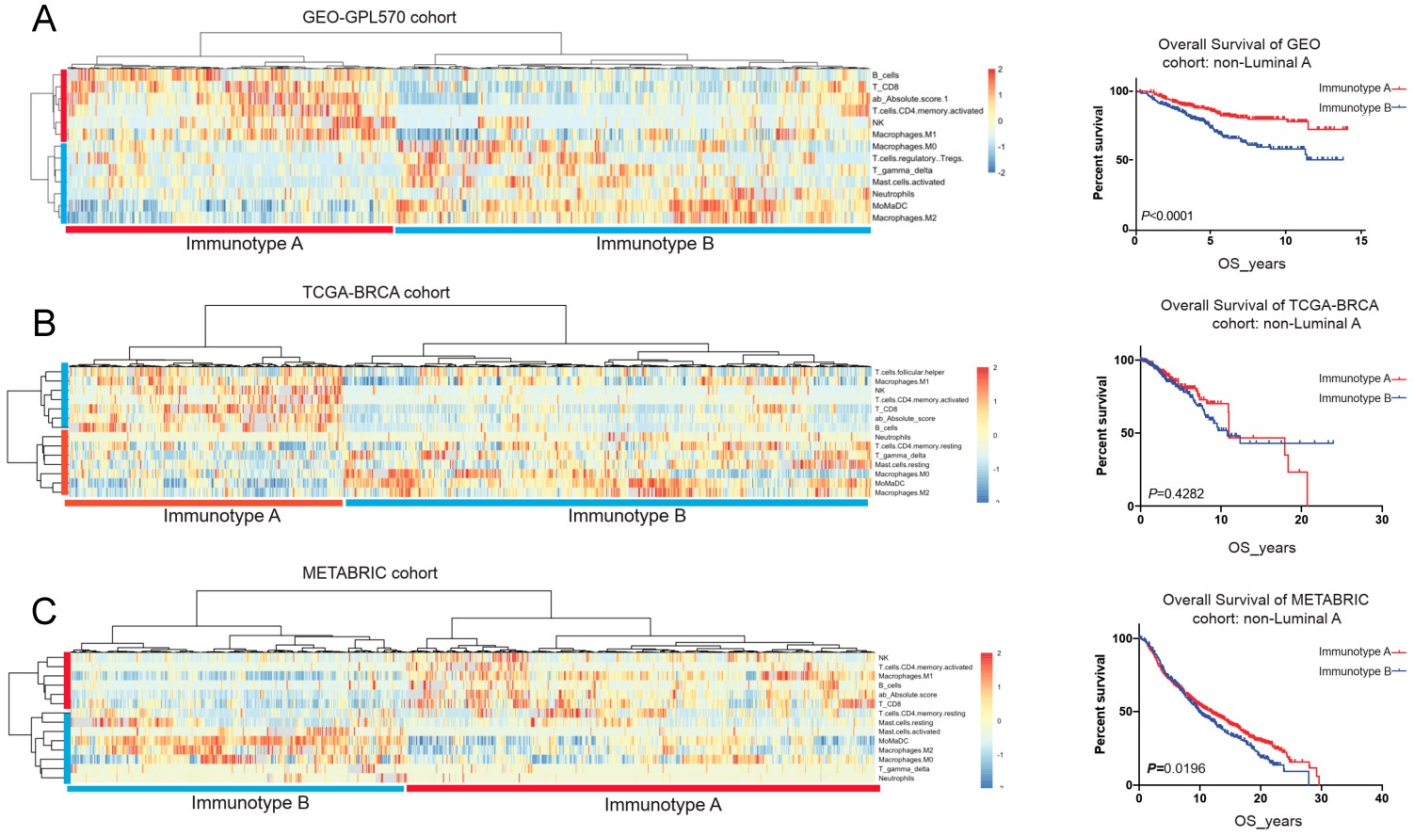
Unsupervised clustering analysis based on immune cell subsets with clinical significance in GEO-GPL570 cohort (A), TCGA-BRCA cohort (B) and METABRIC cohort (C).

Tumour-infiltrating immune cells displayed prognostic significance in luminal B, HER2-enriched and basal-like subtypes, we then performed unsupervised cluster analysis in the above 3 breast cancer molecular types using the GEO cohort. and divided into two immunotypes: immunotype A (immune-reactive) and immunotype B (immune-nonreactive). (Figure 6A) Immunotype A was defined as B_cell^high^ NK^high^ CD8^+^_T^high^ CD4^+^_memory_T_activated^high^ γδT^low^ Mast_cell_activated^low^ Neutrophil^low^, and immunotype B was defined as B_cell^low^ NK^low^ CD8^+^_T^low^ CD4^+^_memory_T_activated^low^ γδT^high^ Mast_cell_activated^high^ Neutrophil^high^. Immunotype A breast cancer had a better prognosis with enrichment of survival-favourable immune cell subsets, whereas immunotype B had a worse prognosis with a higher abundance of survival-unfavourable immune cell subsets. This immunotyping had also been validated in the TCGA-BRCA and METABRIC cohorts and demonstrated that immunotype A had a better prognosis than immunotype B (Figure 6B&C; Table 3).

**Figure 7:**
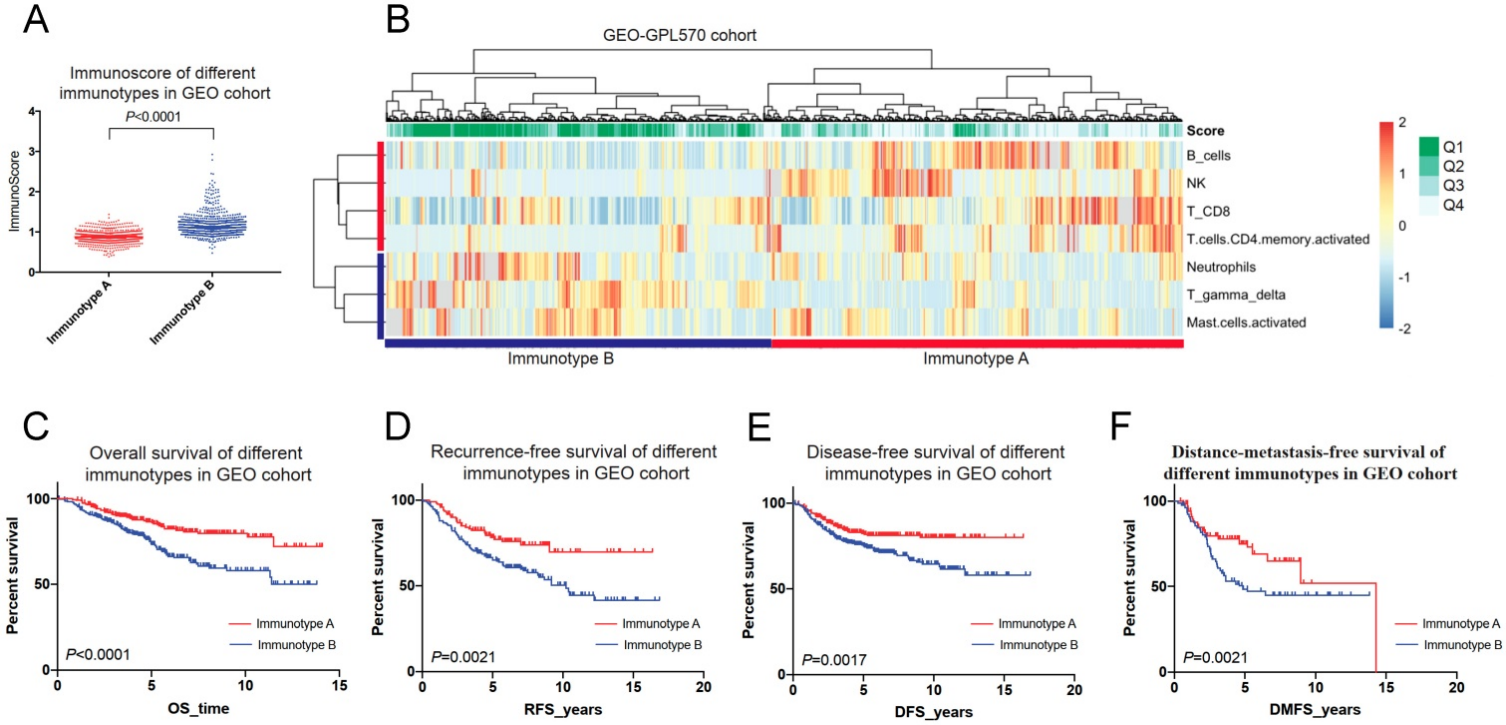
Immunotyping of breast cancer based on the unsupervised clustering analysis of immune cell subsets (A&B) and related prognosis(overall survival, recurrence-free survival, disease-free survival and distant-metastasis-free survival) of immunotype A and B in the GEO cohort (C-F).

In the GEO cohort, immunotype A had more survival favourable cell subsets such as B cells, NK cells, CD8+ T cells and CD4+ memory T cells (Figure 7A-B), and was associated with a better 5-year OS than immunotype B (85.7% *vs*. 73.4%*, P* < 0.001) (Figure 7C). A similar trend was found for RFS, DFS, and DMFS (Figure 7D-F).

**Figure 8:**
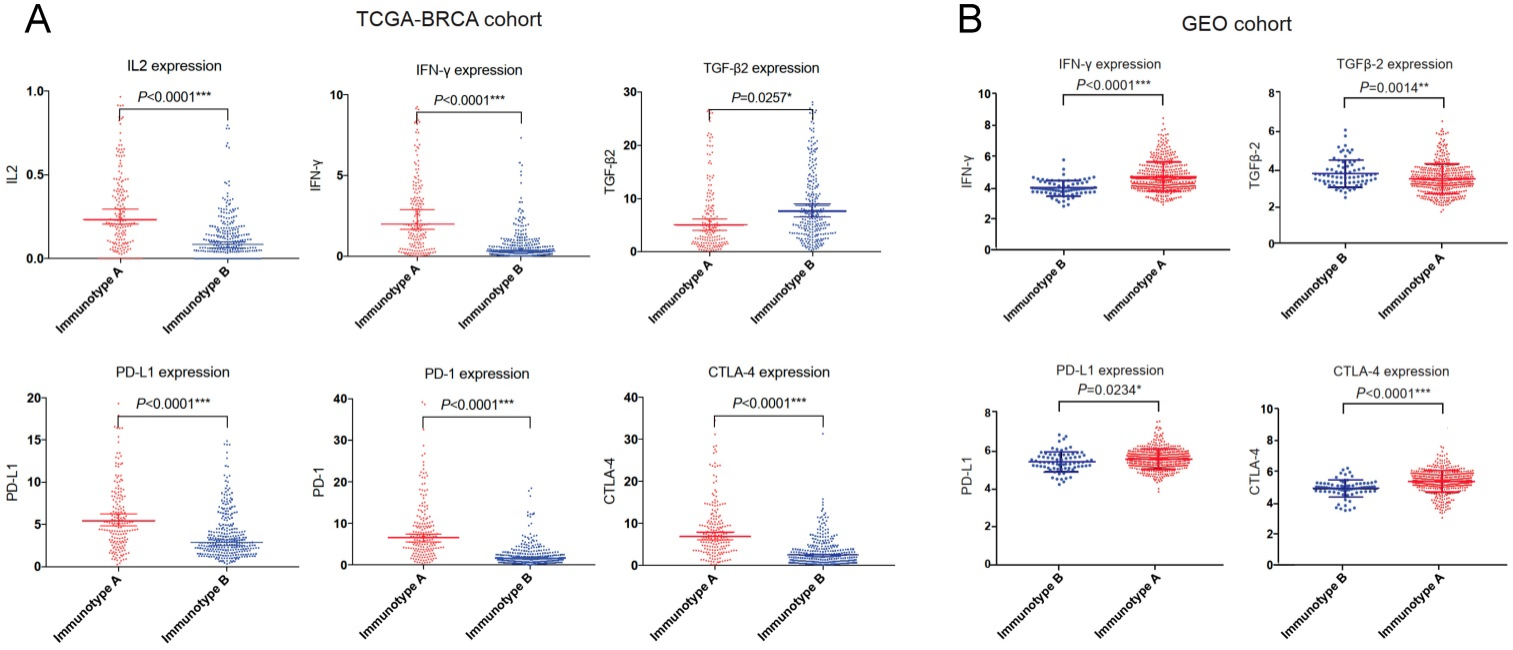
Important immune cytokines and checkpoint molecules in TCGA-BRCA (A) and GEO (B) cohorts.

We also compared the expression of several important cytokines (interleukin 2 (IL-2), interferon γ (IFN-γ), transforming growth factor β (TGF-β) and immune checkpoint molecules (programmed cell death 1 ligand 1 (PD-L1), programmed cell death 1 (PD-1), cytotoxic T lymphocyte antigen 4 (CTLA-4)) in the GEO cohort as well as TCGA-BRCA cohort. IL-2 and IFN-γ expressions were significantly higher in immunotype A, while TGF-β expression was significantly higher in immunotype B. For immune checkpoint molecules, PD-L1, PD-1 and CTLA-4 levels were significantly higher in immunotype A than in immunotype B (Figure 8).

### Immune signature gene analysis between immunotype A and B

We further performed a series of analyses to identify novel differentiated immune signature genes between immunotype A and immunotype B from the GEO, TCGA-BRCA and METABRIC cohorts. A total of 202 immune-related genes with higher expression were identified. KEGG (Kyoto Encyclopaedia of Genes and Genomes) analysis showed related signalling pathways, including T cell differentiation, NK cell toxicity, cytokine-cytokine receptor interaction, NF-κB signalling pathway, etc. (Figure S6). Protein-protein interaction (PPI) analysis demonstrated a network consisting of T cell-, B cell- and NK cell-related genes and cytokines (Figure S7).

To further screen prognosis-related immune signature gene, we performed univariable and multivariable Cox survival analysis for each differentially expressed gene and identified factors significantly correlated with OS. We finally identified Prostaglandin D2 Synthase (PTGDS or lipocalin-type prostaglandin D synthase, L-PGDS) as a novel survival-related immune signature gene (Table S3). There existed rare studies on the biological function of PTGDS in breast cancer. Therefore, we analysed the possible role of PTGDS in breast cancer through bioinformatics analysis and histological evaluation.

**Figure 9:**
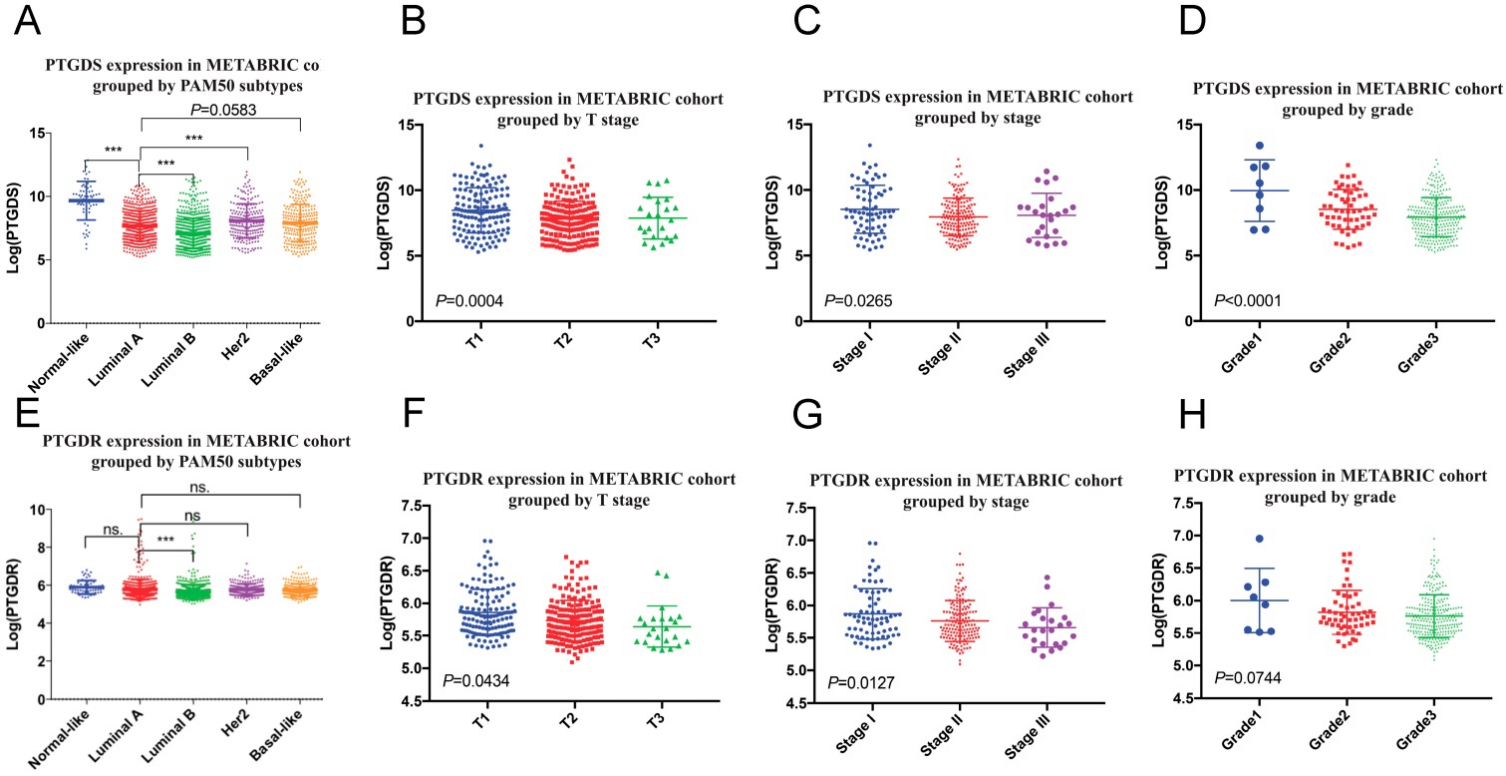
PTGDS (A-D) and PTGDR (E-H) mRNA expression grouped by clinicopathological features in METABRIC cohort. PTGDS, prostaglandin D2 synthase; PTGDR, prostaglandin D2 receptor.

We found that the mRNA levels of PTGDS and its receptor prostaglandin D2 receptor (PTGDR) were downregulated in tumours with larger size, higher stage, and higher histological grade, suggesting that PTGDS could serve as a protective factor (Figure 9). A differential gene analysis was performed between the high and low PTGDS expression groups (divided by mean PTGDS mRNA level), which indicated that PTGDS was positively correlated with immune-related pathways in breast cancer, including the lymphocyte transmembrane migration pathway, T cell signalling pathway, B cell signalling pathway and NK cell-mediated cytotoxicity (Figure S8A). At the same time, we calculated correlation coefficients between the mRNA expression level of PTGDS and the estimated immune cell subsets in the GEO cohort. PTGDS was positively correlated with immune cell subsets estimated by CIBERSORT such as B cells, CD8^+^ T cells, and CD4^+^ T cells and negatively correlated with immune cell subsets such as granulocytes and M0/M2 macrophages, which are unfavourable for survival (Figure S8B).

**Figure 10:**
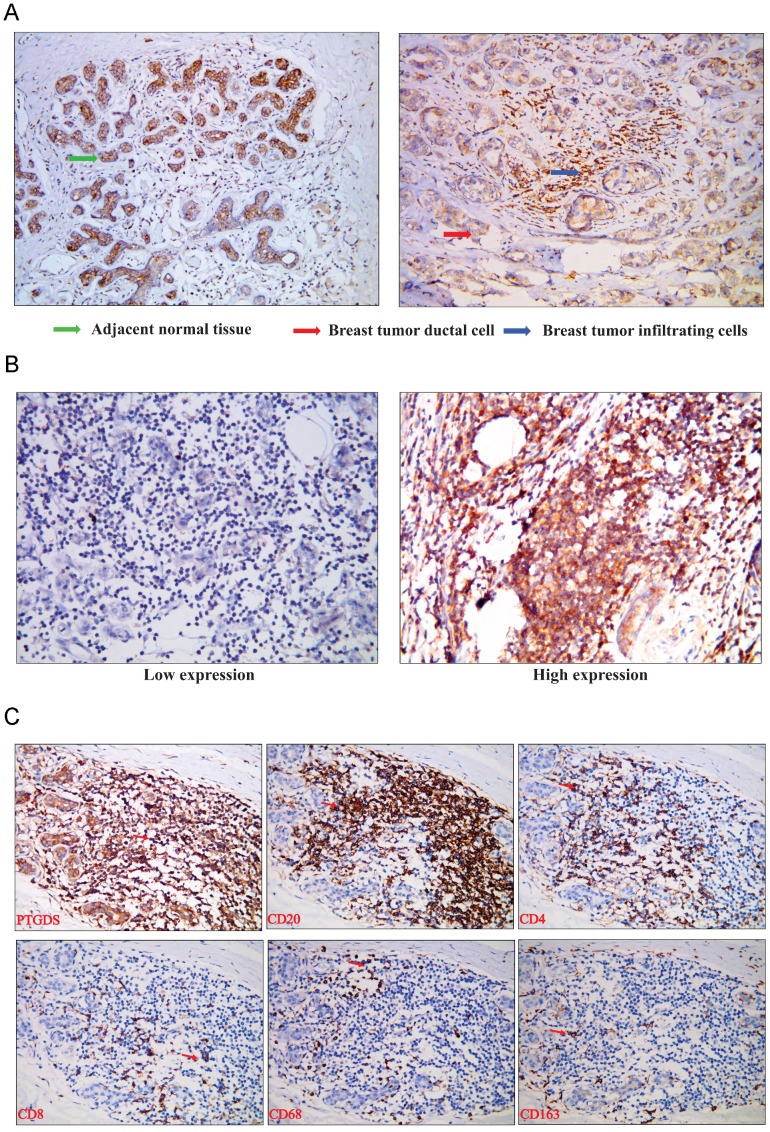
(A) PTGDS expression in a paraffin-embedded TNBC tissue using IHC. (B) PTGDS is expressed heterogeneously in breast cancer tissues based on IHC. (C) co-expression of PTGDS with markers of different immune cell subsets in a TNBC breast cancer tissue. IHC, immunohistochemistry; TNBC, triple-negative breast cancer; PTGDS, prostaglandin D2 synthase.

To analyse the expression of PTDGS in breast tissue, we performed immunohistochemical detection in paraffin-embedded specimens from 98 breast cancer patients with clinicopathological and survival information. The expression of PTGDS was significantly higher in stromal TILs than in ductal epithelial cells in breast cancer specimens, consistent with the results of bioinformatics analysis (Figure 10A). PTGDS was expressed heterogeneously in breast cancer tissues and both nuclear and cytoplasmic localization of PTGDS could be observed (Figure 10B).

**Figure 11:**
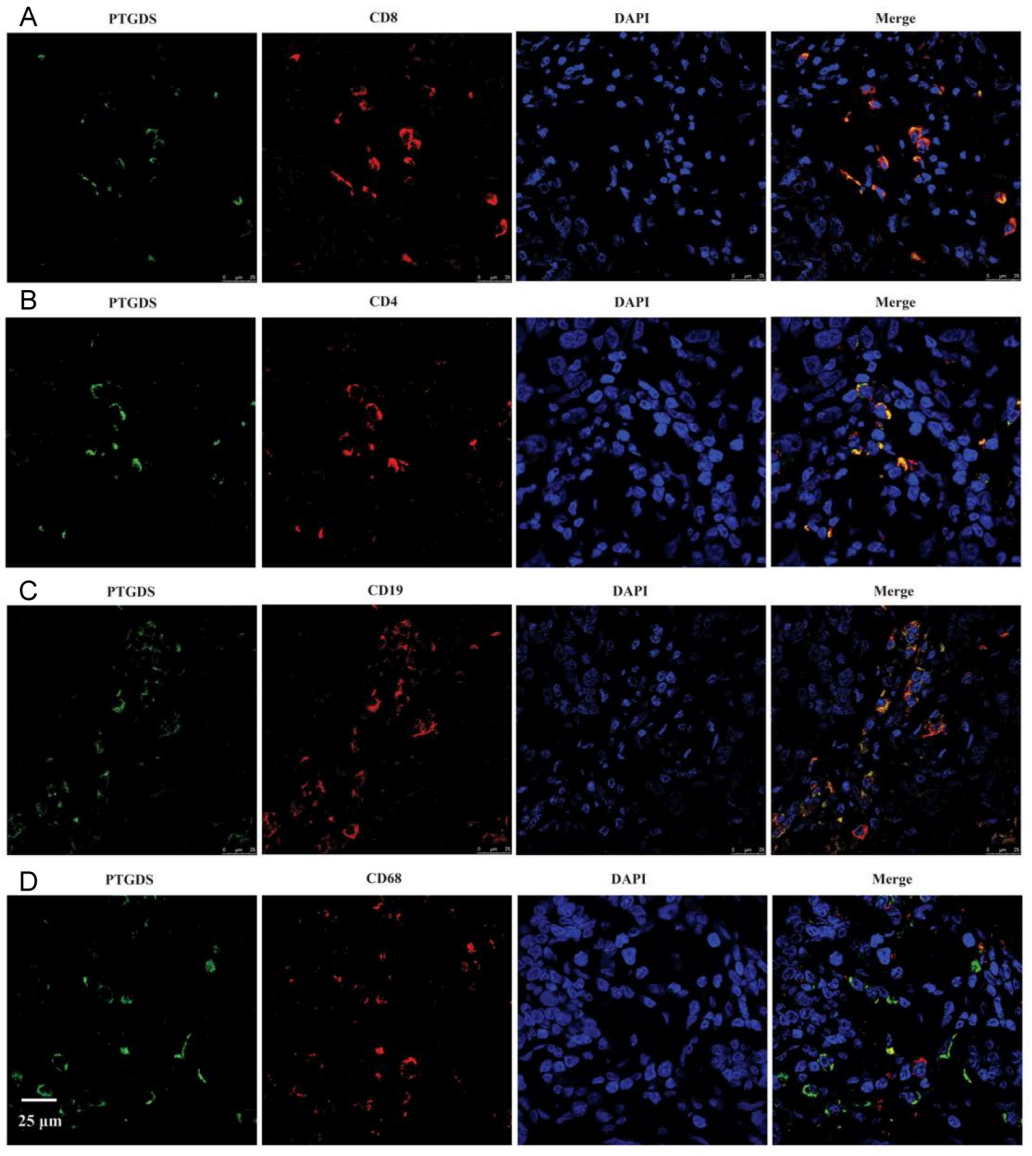
PTGDS expression and co-localization with CD19+ B cells and CD4+/CD8+ T cells in paraffin-embedded TNBC tissues using IF. IF, immunofluorescence; TNBC, triple-negative breast cancer; DAPI, 4',6-diamidino-2-phenylindole; PTGDS, prostaglandin D2 synthase.

To identify the specific cell types expressing PTGDS, we performed IHC staining on serial sections and IF staining in paraffin-embedded breast cancer tissues to detect the localization of PTGDS in different subsets of TILs. The results showed that both CD19^+^/CD20^+^ B cells and CD4^+^/CD8^+^ T cells were co-localized with PTGDS staining (Figure 10C: IHC results; Figure 11: IF results).

The expression level of PTGDS in breast cancer tissues was identified by IHC. We divided all 98 patients into high-expression and low-expression groups based on the average PTGDS expression level. The results indicated that higher expression of PTGDS was related to higher levels of TIL infiltration, smaller tumours, and earlier pathological stages, which was also consistent with the bioinformatics analysis results (Table 4).

## Discussion

The role of immune cells in tumour microenvironment has attracted plenty of attention in recent years. Previous studies focused on the significance of one certain subset in tumour microenvironment but failed to investigate the whole immune cell landscape. The interactions between immune cells in the microenvironment and their ultimate effect on patient prognosis are difficult to validate. Bioinformatics-based genomic integration analysis has brought new opportunities for immune cell landscape research 22. We can use transcriptome data to analyse the abundance of tumour-infiltrating immune cells by deconvolution methods such as CIBERSORT. For breast cancer, a variety of pro- or anti-tumorigenic immune cell subsets are distributed in the same microenvironment, and the total effect is the result of all immune cell combinations, such as CD8+ T and NK cells having anti-tumour activity and Treg cells having tumour-promoting activity 23. This study comprehensively analysed the effects of 22 immune cells in breast cancer microenvironment and established an immune cells-based prognostic model and immunotyping. On this basis, further differential gene analysis between immunotypes revealed that PTGDS plays an important role in mediating local immune response, and the high expression of PTGDS suggests improved prognosis.

Most previous studies focused on the prognostic effect of individual cell subsets 24-26. Ali et al. confirmed a positive correlation between B cells and prognosis and clustered breast cancer patients into 8 types according to immune cell subsets distribution 25. However, these previous studies did not perform further screening of immune cell subsets or establish related prognostic models.

After screening prognostic related immune subsets, our study established a tumour-infiltrating immune cell-based prognostic model in luminal B, HER2-enriched and basal-like breast cancer for the first time. The model prediction results IRS could serve as a novel prognostic marker for poor prognosis. According to the differences in the model coefficient weights, neutrophils and CD4+ memory T cells played a more important role in the microenvironment than other immune cell subsets. As previous reported, increased neutrophils had a negative effect on prognosis 27, while increased CD4+ memory T cells had a positive effect on prognosis 28. CD4+ memory T cells played a more significant role in promoting good prognosis than CD8+ T cells and NK cells, which may be contributed to its role in local immune response activation 29. However, the role of CD4+ memory T cell in tumour microenvironment is still poorly understood, and more studies are needed. The negative effect of neutrophils on prognosis has received a lot of attention in the past two years and targeting tumour-associated neutrophils may be the key to reversing the pro-tumour immune microenvironment 30. The model suggested γδT cells could impair the prognosis, which may be related to the interaction between γδT cells and neutrophils to promote breast cancer metastasis 31. As an important component of TILs, B cell infiltration had been considered as a favourable prognosis marker in breast cancer 32.

After further unsupervised clustering, we divided the included cases into immunotype A (immune-reactive) and immunotype B (immune-nonreactive) in luminal B, HER2-enriched and basal-like subtypes. Immunotype A cases had better 5- and 10-year OS and RFS rates than immunotype B cases, suggesting that immunotyping could be used as a novel independent prognostic tool. Immunotype A was associated with anti-tumour effector cell subsets, such as NK cells, B cells, and T cells, which could explain why patients with immunotype A had a better prognosis than patients with immunotype B. In the METABRIC cohort, the immunotype B cases also had a worse prognosis than immunotype A cases. This is the first tumour infiltrating immune cells-based breast cancer subtyping with clinical significance which only needs tumour tissue transcriptome data. In luminal breast cancer subtypes, OncotypeDX was important for chemotherapy efficacy and patient's prognosis predicting 33. We hope this immunotyping could play a similar role in non-luminal (HER2-enriched and basal-like) breast cancer subtypes in the future.

Based on the survival analysis of immune-related differential genes between immunotype A and B, we identified a novel immune signature gene, lipocalin-type prostaglandin D2 synthase (PTGDS), which was positively correlated with better prognosis. The main function of PTGDS is to convert prostaglandin H2 (PGH2) to prostaglandin D2 (PGD2) 34. Taketomi et al. found that PTGDS could mediate mast cell maturation via PGD2 35. Some studies suggested that PGD2 could induce lymphocyte aggregation with pro-inflammatory effects, but other studies reported that prostaglandin D2 had anti-inflammatory effects by inhibiting dendritic cells and neutrophil aggregation 36. In recent years, studies had reported that PGD2 can inhibit tumour cell growth by inhibiting angiogenesis in the tumour microenvironment 37. As the main synthetase of PGD2, PTGDS had also been shown to be downregulated in multiple tumours, such as lung cancer 38, gastric cancer 39, prostate cancer 40, and cervical cancer 41. However, the detailed molecular mechanism is still not clear. PTGDS was highly expressed in metastatic lymph nodes, suggesting PTGDS was associated with an immune response 42, 43. Lipocalin 2 (LCN2), which belonged to the lipocalin superfamily the same as PTGDS, had been widely studied in various tumours. LCN2 was up-regulated by endoplasmic reticulum (ER) stress response in hypoxia and pro-inflammatory tumour microenvironment and could promote epithelial-to-mesenchymal transition (EMT), which contributing to cancer cell invasiveness 44, 45. The lipocalin superfamily might have an important role in tumour immune-related microenvironment transformation.

We explored the biological functions and signalling pathways PTGDS by bioinformatics analysis. KEGG pathway enrichment analysis suggested that PTGDS might play a role in immune response, cytokine interaction, T cell signalling, NK-mediated cytotoxicity, etc. IHC analysis of paraffin-embedded clinical breast cancer specimens demonstrated that PTGDS was positively correlated with better clinicopathological features. The expression of PTGDS coincided with TILs. Further colocalization experiments demonstrated that PTGDS was highly expressed in CD19^+^ B cells, CD4^+^ T cells and CD8^+^ T cells, suggesting that its protective effect may be enhanced by the anti-tumour effects of B cells and T cells. However, the specific molecular mechanism underlying the effect of PTGDS on lymphocyte maturation and function remains to be confirmed by further studies.

There are still some limitations in this study. First, the bioinformatics method used to evaluate immune cells in breast cancer tissues could not accurately discriminate immune cells across specific spatial locations, such as intrastromal/intratumoural or invasive tumour margin/tumour centre. Traditional methods such as H&E staining, IHC, and IF can help to compensate for this deficiency. Second, the standard therapy of breast cancer varies across different databases by different regions and years. These factors may cause disturbance in the nonlinear relationship between IRS and OS.

In this study, we established a novel immunotyping and a tumour-infiltrating immune cell-based breast cancer prognostic prediction model by analysing the prognostic significance of multiple immune cell subsets in luminal B, HER2-enriched and basal-like breast cancer for the first time. These results could not only serve as a tool for prognostic prediction but also provide potential information for individualized treatment. Based on gene screening between immunotypes A and B, a novel breast cancer immune signature gene PTDGS was discovered, and the expression pattern of PTGDS in the breast cancer microenvironment was identified, which suggested that PTDGS may play an important role in breast cancer development and lymphocyte-related immune response and thus serve as a potential target for breast cancer diagnosis and treatment.

## Supplementary Material

Supplementary figures and tables.

## Figures and Tables

**Table 1:** Clinical information of included breast cancer patients

Cohort		GEO	TCGA	METABRIC
**N**		1680	635	480
**Age, years old**		52.9±12.9	57.2±12.7	58.6±13.1
**T stage**	T1	423	163	223
	T2	770	368	225
	T3	196	82	25
	T4	24	23	NA
**N stage**	N0	558	287	NA
	N1-N3	715	348	NA
**M stage**	M0	310	608	367
	M1	5	17	1
**PAM50 subtype**	Normal-like	87	21	21
	Luminal A	463	129	87
	Luminal B	452	276	152
	HER2-enriched	284	76	100
	Basal-like	394	133	120
**Grade**	I	158	NA	16
	II	319	NA	133
	III	573	NA	322

OS, overall survival; RFS, recurrence-free survival; DFS, disease-free survival; DMFS, distant-metastasis-free survival; NA=not avaiable.

**Table 2:** Clinical significance of different immune cell subsets

Immune Cell Subsets	HR	95% CI	*p*
Survival favourable cell subsets			
B cells	0.05	0.005-0.454	0.008^**^
CD8^+^ T cells	0.12	0.025-0.562	0.007^**^
Activated CD4^+^ memory T cells	6.56e-05	0.000-0.022	0.001^**^
M1 macrophages	0.01	0.001-0.341	0.009^**^
NK cells	2.80e-06	0.000-0.018	0.004^**^
Survival unfavourable cell subsets			
Treg cells	745.60	1.489-3.73e+5	0.037^*^
M0 macrophages	2.28	0.462-11.28	0.311
M2 macrophages	13.06	1.785-95.58	0.011^*^
Activated mast cells	203.10	0.878-4.69 e+4	0.056
Neutrophils	1.69e+10	1.27e+5-2.24e+15	9e-05^***^
γδT cells	645.30	29.010-1.44 e+4	4e-05^***^
Neutral cell subsets			
Dendritic cells	0.03	0.000-122.60	0.396
Eosinophils	18.04	0.001-2.55 e+5	0.553
T follicular helper cells	14.14	0.187-1072	0.230

HR, hazard ratio; CI, confidence interval; ^*^P < 0.05, ^**^P < 0.01, ^***^P < 0.001.

**Table 3:** Multivariable survival analysis in luminal B/HER2-enriched/basal-like breast cancer patients (N=342, events=92)

Variable	HR	95% CI	*P*-value
Age at diagnosis			
T stage (T1 *vs*. T2-T4)	0.05	0.01-0.16	<0.001***
N stage (N0 *vs*. N1-N3)	0.05	0.01-0.45	0.008**
PAM50 subtype	0.12	0.03-0.56	0.007**
Immunotype (Immunotype A *vs*. Immunotype B)	< 0.01	0.00-0.02	0.001**

HR, hazard ratio; CI, confidence interval; *P < 0.05, **P < 0.01, ***P < 0.001.

**Table 4:** Clinical and pathological characteristics of PTGDS ^high^ and PTGDS ^low^ group

Variables	PTGDS ^high^	PTGDS ^low^	*P*-value
Number of cases	41 (40.8%)	57 (59.2%)	
Age at diagnosis (years)	51.4 ± 7.8	52.5 ± 9.4	0.65
BMI (kg/m^2^)	23.12 ± 3.10	25.59 ± 4.25	0.01**
Breast-conserving surgery	18 (43.9%)	32 (56.1%)	0.32
ALD	10 (24.4%)	20 (35.1%)	0.36
Molecular subtype			0.04*
Luminal A	3 (7.3%)	4 (7.0%)	
Luminal B	10 (24.4%)	26 (45.6%)	
Her2	8 (19.5%)	14 (24.6%)	
TNBC	20 (48.8%)	13 (22.8%)	
Ki-67	0.37	0.39	0.88
T stage			0.04*
I	32 (78.0%)	32 (56.1%)	
II-III	9 (22.0%)	25 (43.9%)	
N stage			0.54
N0	33 (80.5%)	41 (71.9%)	
N1-N3	8 (19.5%)	16 (28.1%)	
Pathological stages			0.11
I	24 (58.5%)	24 (42.1%)	
II	16 (39.0%)	26 (45.6%)	
III	1 (2.4%)	7 (12.3%)	
Histological grade			0.55
G1	2 (4.9%)	1 (1.8%)	
G2	20 (48.8%)	33 (57.9%)	
G3	19 (46.3%)	23 (40.3%)	
Percentage of invasive tumor	0.86 ± 0.23	0.81 ± 0.17	0.44
TILs	0.34 ± 0.23	0.24 ± 0.18	0.12
PTGDS intensity	2.75 ± 0.44	1.34 ± 1.11	<0.0001***

CI, confidence interval; *P < 0.05, **P < 0.01, ***P < 0.001.

## Abbreviations

CI: confidence interval
DAPI: 4',6-diamidino-2-phenylindole
DFS: disease-free survival
DMFS: distant-metastasis-free survival
HR: hazard ratio
IRS: immunorisk score
MoMaDC: macrophages, monocytes and dendritic cells
OS: overall survival
PTGDR: prostaglandin D2 receptor
PTGDS: prostaglandin D2 synthase
RFS: recurrence-free survival
