# Supplementary material for: Tumour-Infiltrating Immune Cell-Based Subtyping and Signature Gene Analysis in Breast Cancer Based on Gene Expression Profiles
Source: J Cancer. 2020 Jan 14;11(6):1568–83. doi: 10.7150/jca.37637 (PMC6995381; doi:10.7150/jca.37637)
Supplement: Supplementary file 1 — Supplementary figures and tables. [file jcav11p1568s1.pdf]

Figure S1. The absolute immune score estimated by CIBERSORT-LM22 grouped by PAM50 subtypes in the GEO cohort.

Figure S2. Relative percentages of cell subsets estimated by CIBERSORT-LM7 (A, C, E, G) and CIBERSORT-LM22 (B, D, F, H) grouped by clinicalpathological features in GEO cohort. MoMaDC, macrophages, monocytes and DCs.

Figure S3. Univariable (A, C, E) and multivariable (B, D, F) Cox regression analysis of cell subsets estimated by CIBERSORT-LM7 or CIBERSORT-LM22 in GEO cohort.

Figure S4. Pearson's correlation coefficients between immune cell subsets with clinical significance in GEO cohort.

Figure S5. Multivariable survival analyses of immunorisk score in OS (A), RFS (B), DFS (C), DMFS (D) in GEO cohort. OS, overall survival; RFS, recurrence-free survival; DFS, disease-free survival; DMFS, distant metastasis-free survival; HR, hazard ratio; CI, confidence interval.

Figure S6. KEGG enrichment analysis based on the differential genes between immunotype A and immunotype B.

Figure S7. Protein-protein interaction network based on the differential genes between immunotype A and immunotype B.

Figure S8. PTGDS-related signalling pathways (A) and immune cell subsets (B) in GEO cohort. PTGDS, prostaglandin D2 synthase.

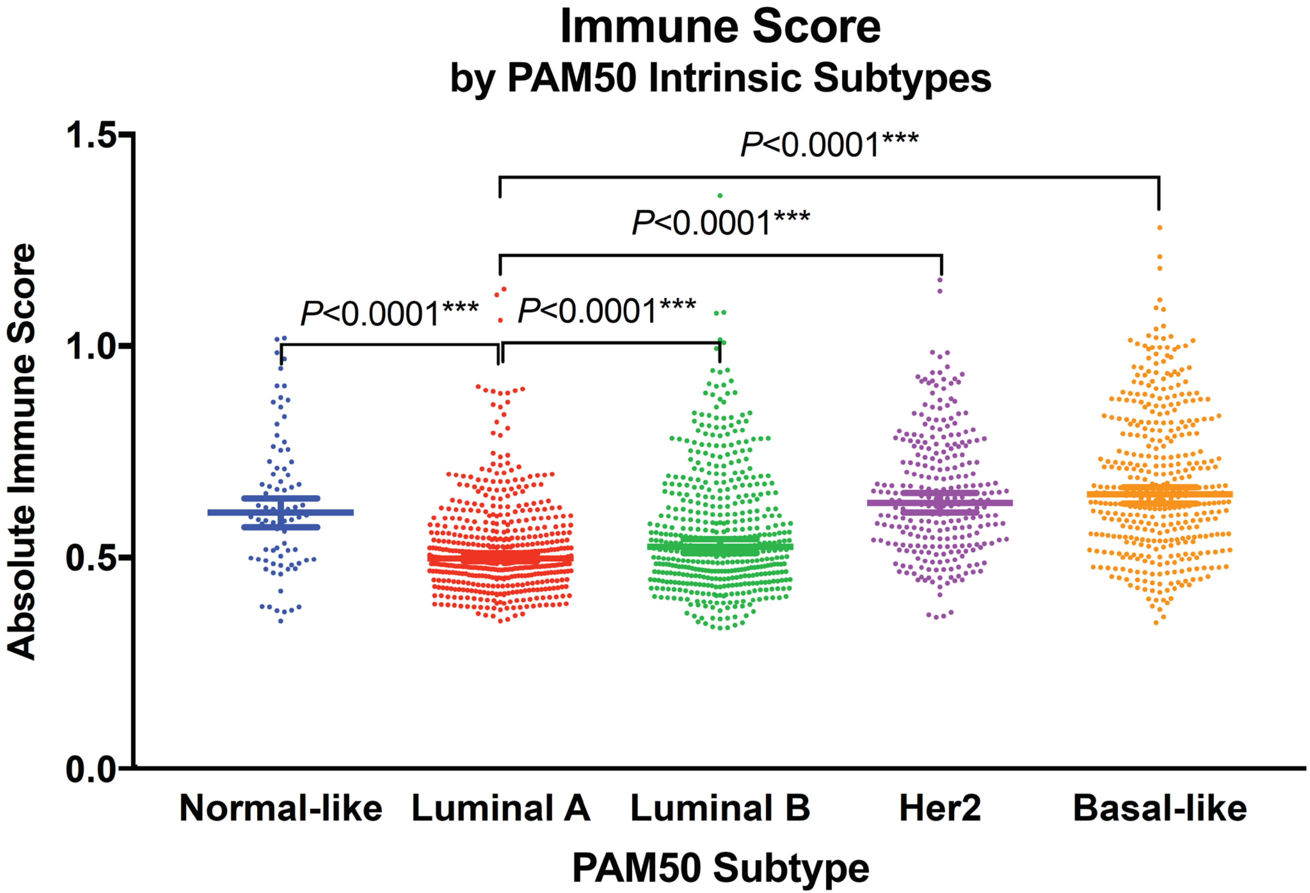
Immune Score
by PAM50 Intrinsic Subtypes
1.5
1.0
0.5
0.0
Absolute Immune Score
P<0.0001***
P<0.0001***
P<0.0001***
P<0.0001***
Normal-like
Luminal A
Luminal B
Her2
Basal-like
PAM50 Subtype

Figure S1

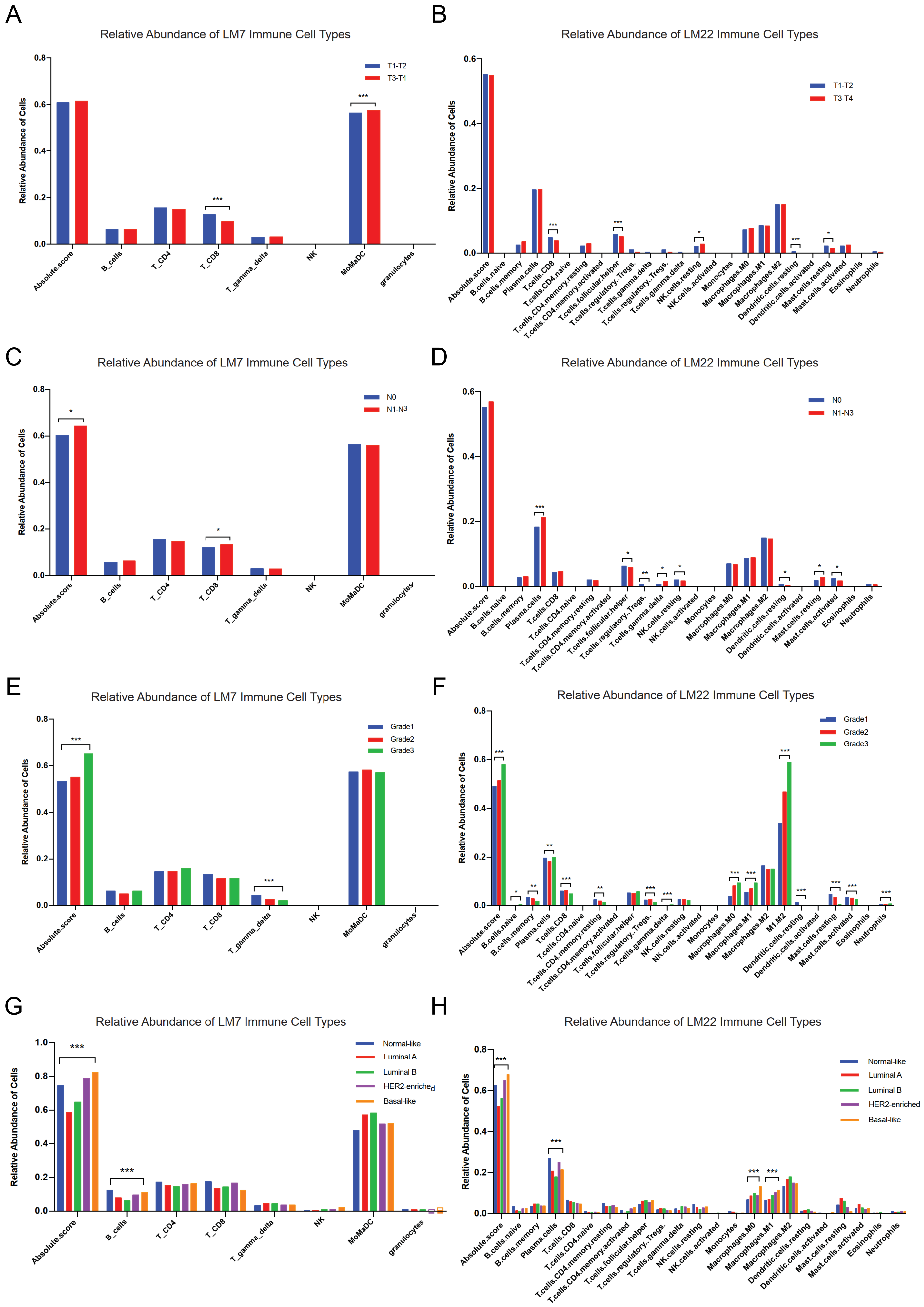

A
Relative Abundance of LM7 Immune Cell Types
B
Relative Abundance of LM22 Immune Cell Types
C
Relative Abundance of LM7 Immune Cell Types
D
Relative Abundance of LM22 Immune Cell Types
E
Relative Abundance of LM7 Immune Cell Types
F
Relative Abundance of LM22 Immune Cell Types
G
Relative Abundance of LM7 Immune Cell Types
H
Relative Abundance of LM22 Immune Cell Types
Relative Abundance of Cells
T1-T2
T3-T4
N0
N1-N3
Grade1
Grade2
Grade3
Normal-like
Luminal A
Luminal B
HER2-enriched
Basal-like
Absolute.score
B_cells
T_CD4
T_CD8
T_gamma_delta
NK
MoMaDC
granulocytes

Figure S2

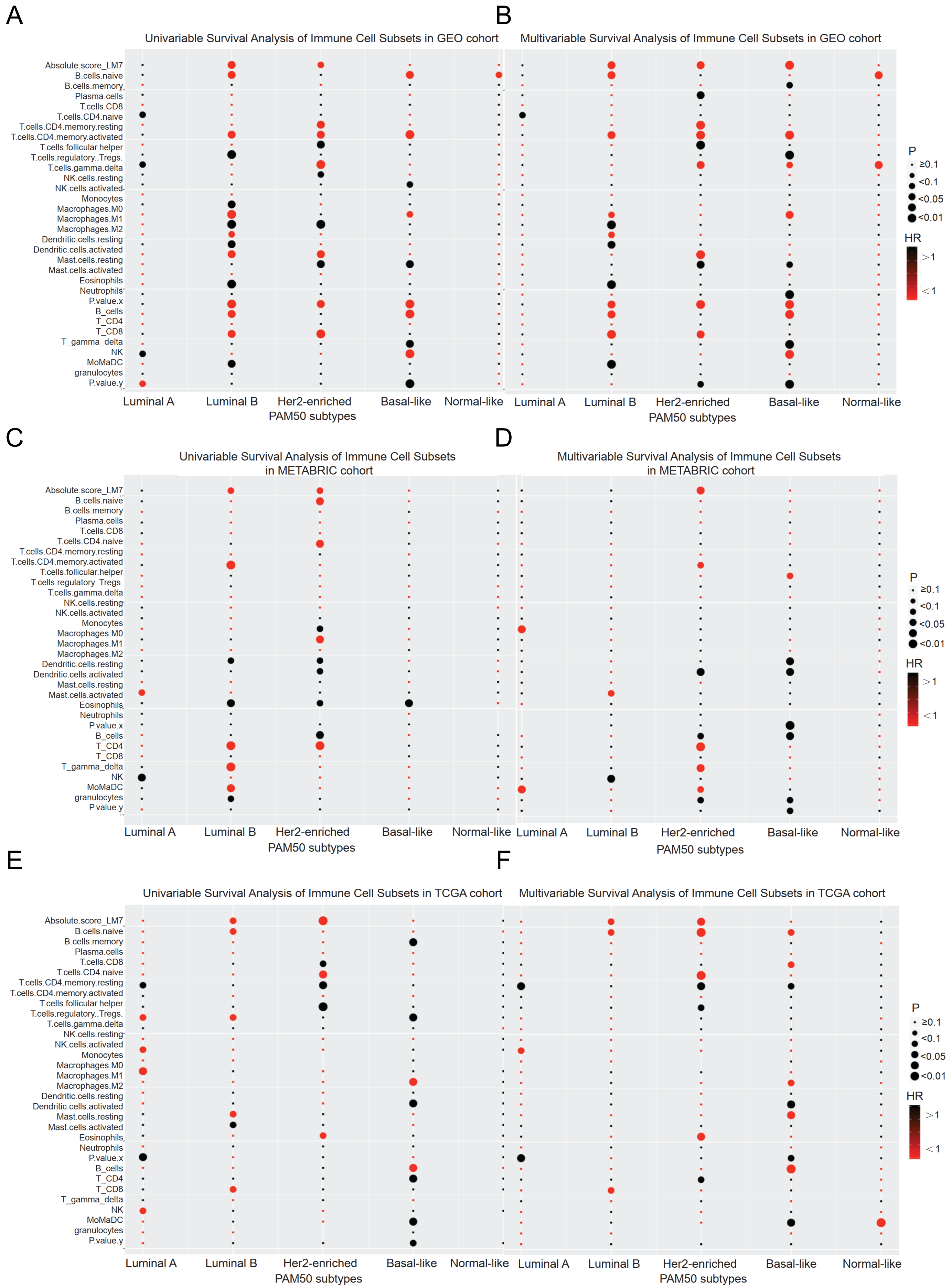
A
Univariable Survival Analysis of Immune Cell Subsets in GEO cohort
B
Multivariable Survival Analysis of Immune Cell Subsets in GEO cohort
Absolute.score_LM7
B.cells.naive
B.cells.memory
Plasma.cells
T.cells.CD8
T.cells.CD4.naive
T.cells.CD4.memory.resting
T.cells.CD4.memory.activated
T.cells.follicular.helper
T.cells.regulatory..Tregs.
T.cells.gamma.delta
NK.cells.resting
NK.cells.activated
Monocytes
Macrophages.M0
Macrophages.M1
Macrophages.M2
Dendritic.cells.resting
Dendritic.cells.activated
Mast.cells.resting
Mast.cells.activated
Eosinophils
Neutrophils
P.value.x
B_cells
T_CD4
T_CD8
T_gamma_delta
NK
MoMaDC
granulocytes
P.value.y
Luminal A
Luminal B
Her2-enriched
Basal-like
Normal-like
PAM50 subtypes
P
≥0.1
<0.1
<0.05
<0.01
HR
>1
<1
C
Univariable Survival Analysis of Immune Cell Subsets in METABRIC cohort
D
Multivariable Survival Analysis of Immune Cell Subsets in METABRIC cohort
E
Univariable Survival Analysis of Immune Cell Subsets in TCGA cohort
F
Multivariable Survival Analysis of Immune Cell Subsets in TCGA cohort

Figure S3

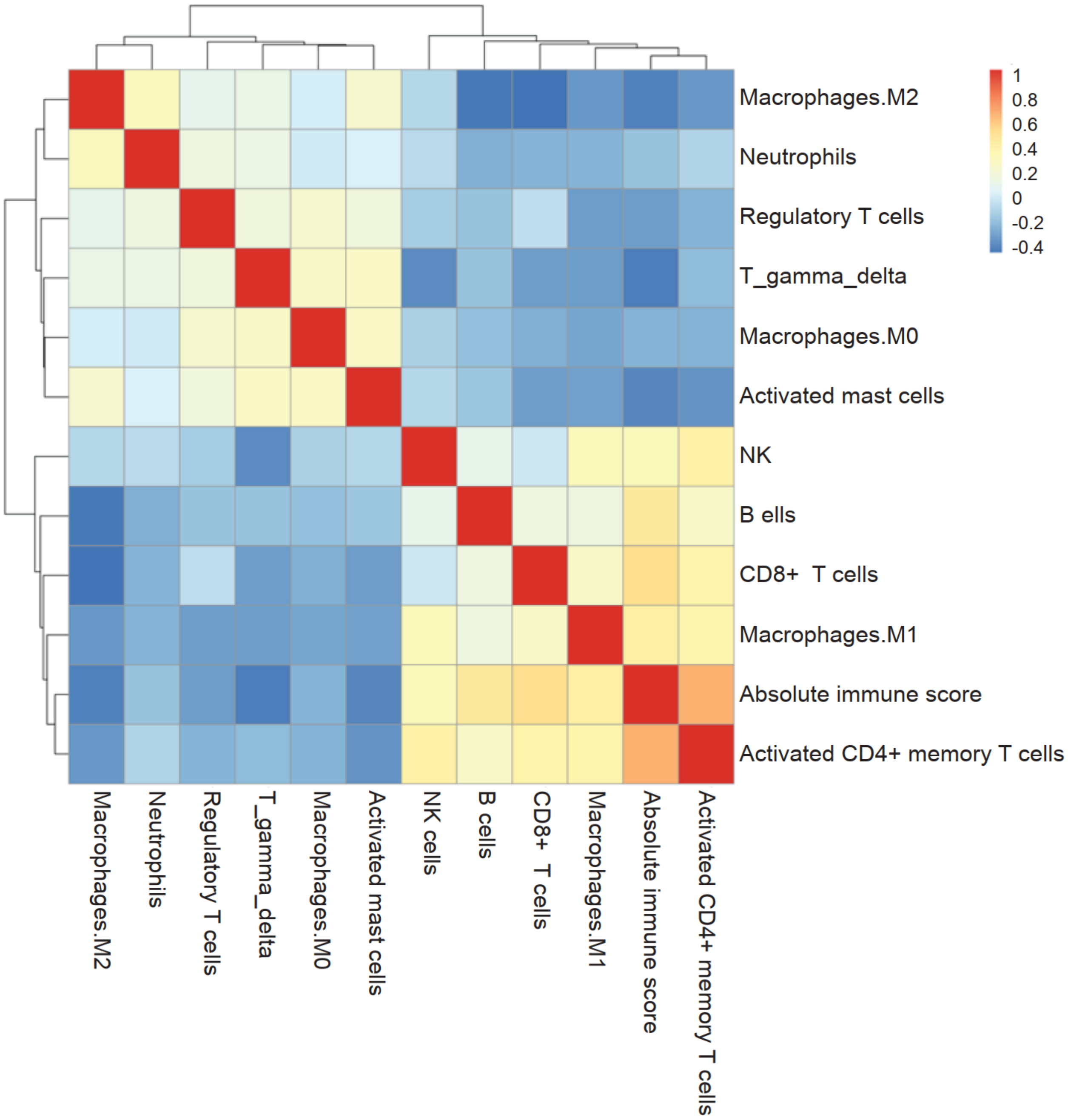
1
0.8
0.6
0.4
0.2
0
-0.2
-0.4
Macrophages.M2
Neutrophils
Regulatory T cells
T_gamma_delta
Macrophages.M0
Activated mast cells
NK
B ells
CD8+ T cells
Macrophages.M1
Absolute immune score
Activated CD4+ memory T cells
Macrophages.M2
Neutrophils
Regulatory T cells
T_gamma_delta
Macrophages.M0
Activated mast cells
NK cells
B cells
CD8+ T cells
Macrophages.M1
Absolute immune score
Activated CD4+ memory T cells

Figure S4

A

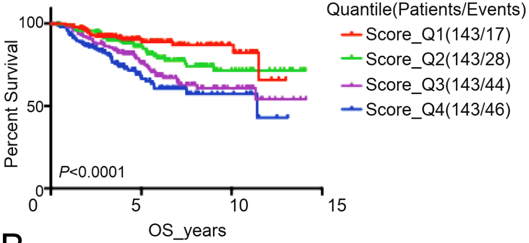

Multivariable survival analyses in non-LuminalA population(N=342, OS events=92)

	HR	95%CI		P
diagnosed age	1.010	0.992	1.028	0.9164
T_stage	1.414	1.009	1.980	0.0144
N_class	2.899	1.721	4.882	0.3877
pam50	1.554	1.202	2.009	0.0333
Score	2.212	1.436	3.406	0.0734

B

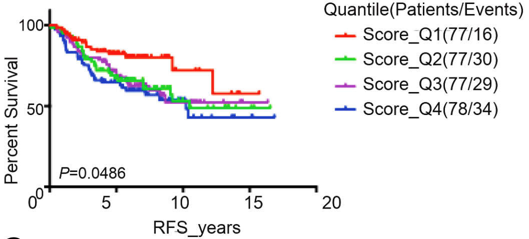

Multivariable survival analyses in non-LumA population(N=202, RFS events=66)

	HR	95%CI		P
diagnosed age	1.010	0.992	1.028	0.2851
T_stage	1.414	1.009	1.980	0.2829
N_class	2.899	1.721	4.882	0.0005
pam50	1.554	1.202	2.009	8.8e-07
Score	2.212	1.436	3.406	0.0049

C

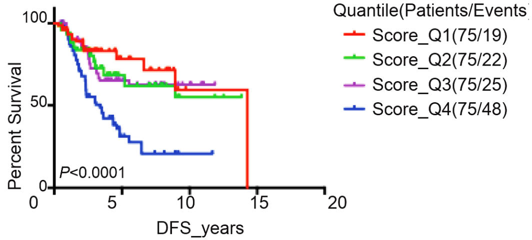

Multivariable survival analyses in non-LumA population(N=145, DFS events=55)

	HR	95%CI		P
diagnosed age	0.994	0.973	1.016	0.6090
T_stage	1.330	0.908	1.945	0.1440
N_class	1.590	0.895	2.811	0.1140
pam50	1.160	0.846	1.582	0.3600
Score	6.120	2.79	13.405	5.9e-06

D

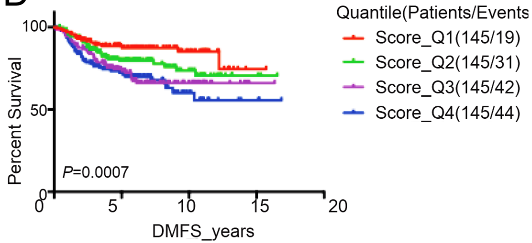

Multivariable survival analyses in non-LumA population(N=342, DMFS events=92)

	HR	95%CI		P
diagnosed age	0.998	0.981	1.015	0.7923
T_stage	1.690	1.204	2.360	0.0024
N_class	2.320	1.436	3.750	0.0006
pam50	1.070	0.819	1.387	0.6364
Score	3.350	2.057	5.463	1.21e-06

Figure S5

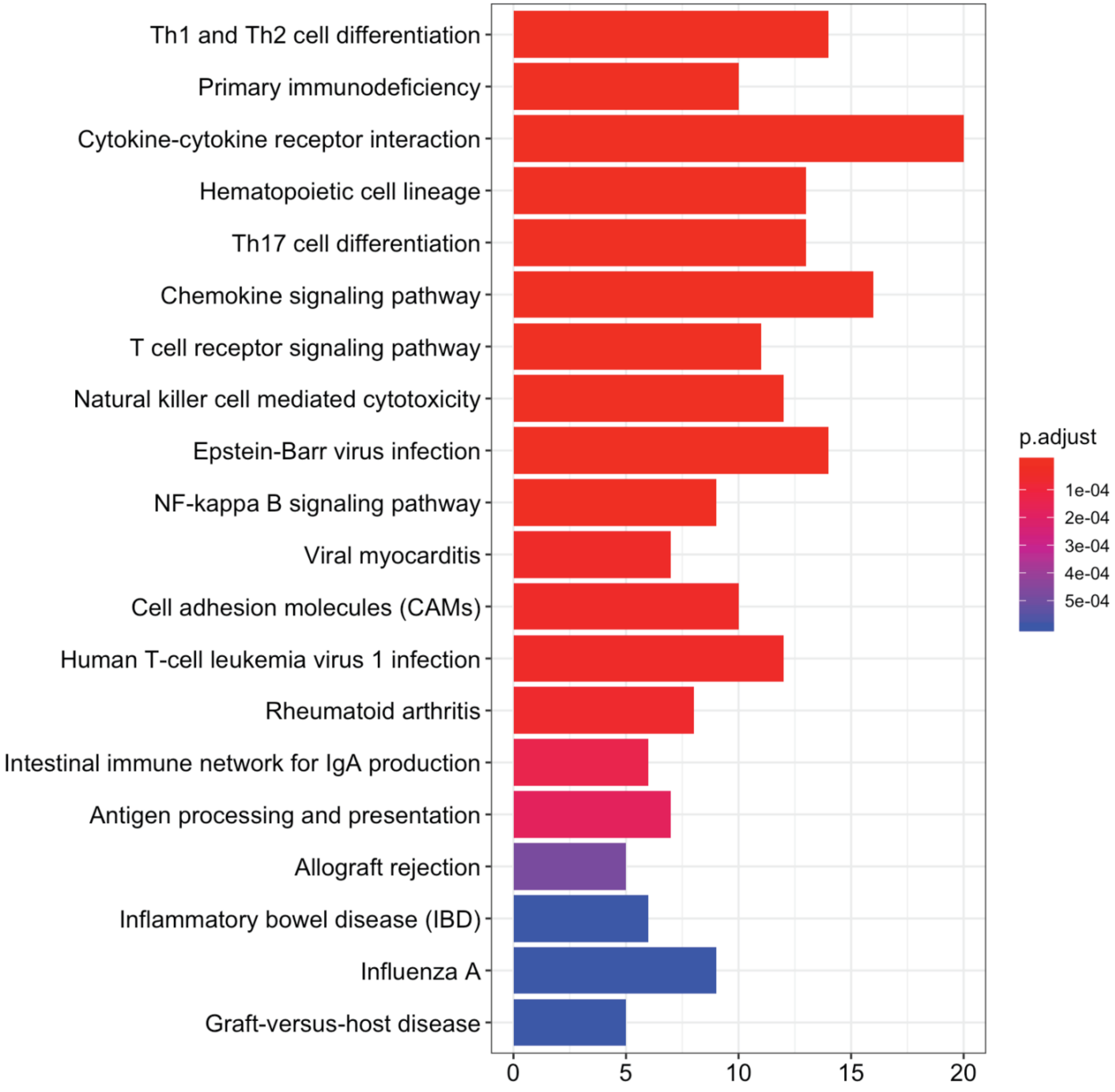
Th1 and Th2 cell differentiation
Primary immunodeficiency
Cytokine-cytokine receptor interaction
Hematopoietic cell lineage
Th17 cell differentiation
Chemokine signaling pathway
T cell receptor signaling pathway
Natural killer cell mediated cytotoxicity
Epstein-Barr virus infection
NF-kappa B signaling pathway
Viral myocarditis
Cell adhesion molecules (CAMs)
Human T-cell leukemia virus 1 infection
Rheumatoid arthritis
Intestinal immune network for IgA production
Antigen processing and presentation
Allograft rejection
Inflammatory bowel disease (IBD)
Influenza A
Graft-versus-host disease
0
5
10
15
20
p.adjust
1e-04
2e-04
3e-04
4e-04
5e-04

Figure S6

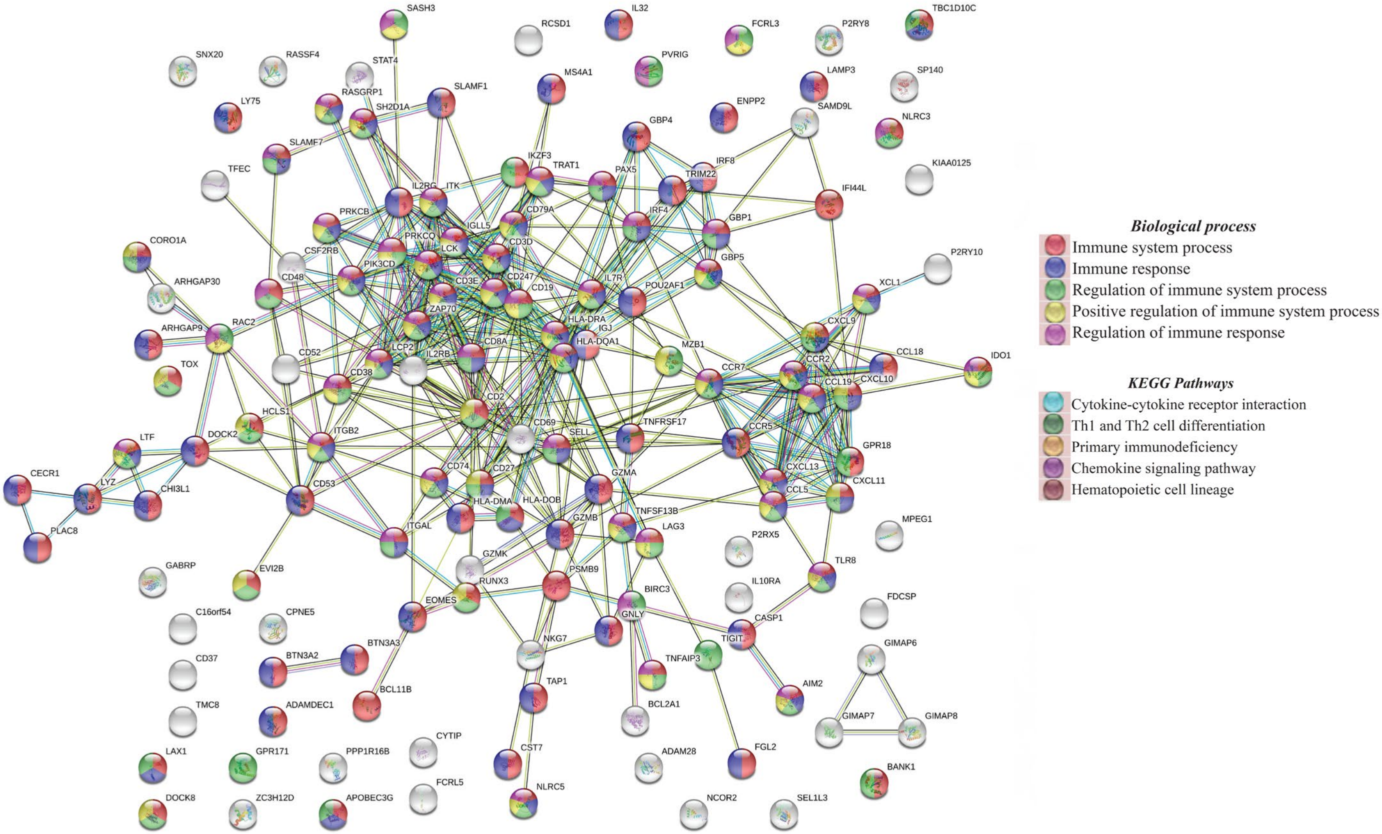
SASH3
IL32
TBC1D10C
RCSD1
FCRL3
P2RY8
SNX20
RASSF4
STAT4
PVRIG
SP140
MS4A1
LAMP3
RASGRP1
SLAMF1
LY75
SH2D1A
ENPP2
SAMD9L
NLRC3
GBP4
SLAMF7
TFEC
IKZF3
TRAT1
KIAA0125
IRF8
PAX5
TRIM22
IFI44L
IL2RG
ITK
PRKCB
CD79A
IRF4
IGLL5
GBP1
PRKCQ
LCK
CD3D
CORO1A
CSF2RB
GBP5
P2RY10
PIK3CD
CD48
CD247
ARHGAP30
CD3E
CD19
IL7R
POU2AF1
XCL1
ZAP70
HLA-DRA
CXCL9
RAC2
ARHGAP9
IGJ
HLA-DQA1
CD8A
MZB1
CCL18
IDO1
CD52
LCP2
IL2RB
CCR2
TOX
CD38
CCR7
CCL19
CXCL10
CD2
HCLS1
CD69
TNFRSF17
SELL
CCR5
LTF
DOCK2
ITGB2
GPR18
CECR1
CD74
CD27
GZMA
CXCL13
CXCL11
LYZ
CHI3L1
CD53
HLA-DMA
HLA-DOB
CCL5
TNFSF13B
MPEG1
PLAC8
ITGAL
GZMB
LAG3
P2RX5
GZMK
GABRP
EVI2B
RUNX3
PSMB9
TLR8
IL10RA
BIRC3
FDCSP
EOMES
C16orf54
CPNE5
GNLY
CASP1
NKG7
TIGIT
BTN3A3
GIMAP6
CD37
BTN3A2
TNFAIP3
TAP1
AIM2
BCL11B
TMC8
ADAMDEC1
BCL2A1
GIMAP7
GIMAP8
CYTIP
LAX1
GPR171
PPP1R16B
CST7
ADAM28
FGL2
BANK1
FCRL5
NLRC5
DOCK8
ZC3H12D
APOBEC3G
NCOR2
SEL1L3
Biological process
Immune system process
Immune response
Regulation of immune system process
Positive regulation of immune system process
Regulation of immune response
KEGG Pathways
Cytokine-cytokine receptor interaction
Th1 and Th2 cell differentiation
Primary immunodeficiency
Chemokine signaling pathway
Hematopoietic cell lineage

Figure S7

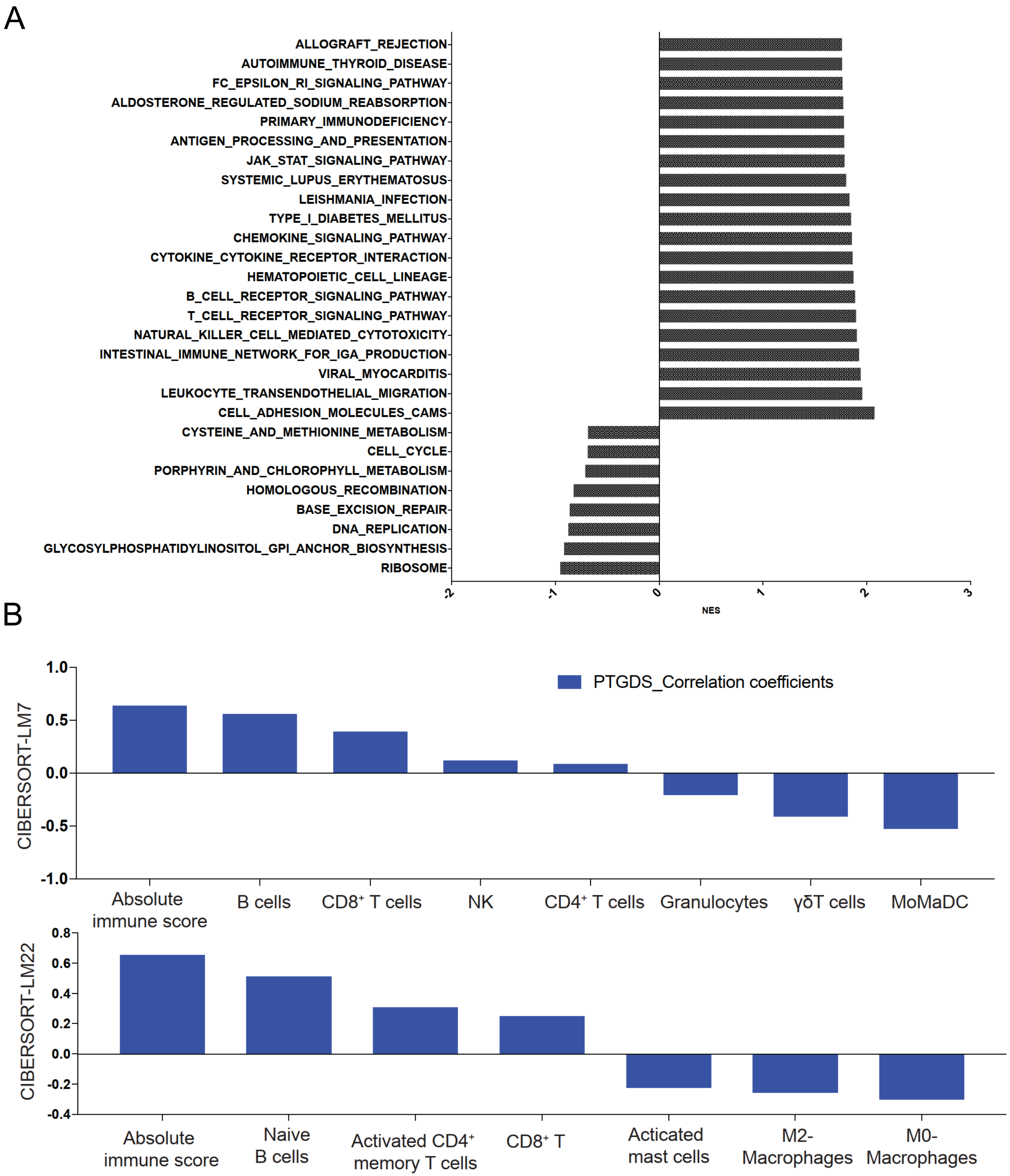
A
ALLOGRAFT_REJECTION
AUTOIMMUNE_THYROID_DISEASE
FC_EPSILON_RI_SIGNALING_PATHWAY
ALDOSTERONE_REGULATED_SODIUM_REABSORPTION
PRIMARY_IMMUNODEFICIENCY
ANTIGEN_PROCESSING_AND_PRESENTATION
JAK_STAT_SIGNALING_PATHWAY
SYSTEMIC_LUPUS_ERYTHEMATOSUS
LEISHMANIA_INFECTION
TYPE_I_DIABETES_MELLITUS
CHEMOKINE_SIGNALING_PATHWAY
CYTOKINE_CYTOKINE_RECEPTOR_INTERACTION
HEMATOPOIETIC_CELL_LINEAGE
B_CELL_RECEPTOR_SIGNALING_PATHWAY
T_CELL_RECEPTOR_SIGNALING_PATHWAY
NATURAL_KILLER_CELL_MEDIATED_CYTOTOXICITY
INTESTINAL_IMMUNE_NETWORK_FOR_IGA_PRODUCTION
VIRAL_MYOCARDITIS
LEUKOCYTE_TRANSENDOTHELIAL_MIGRATION
CELL_ADHESION_MOLECULES_CAMS
CYSTEINE_AND_METHIONINE_METABOLISM
CELL_CYCLE
PORPHYRIN_AND_CHLOROPHYLL_METABOLISM
HOMOLOGOUS_RECOMBINATION
BASE_EXCISION_REPAIR
DNA_REPLICATION
GLYCOSYLPHOSPHATIDYLINOSITOL_GPI_ANCHOR_BIOSYNTHESIS
RIBOSOME
-2
-1
0
1
2
3
NES
B
PTGDS_Correlation coefficients
CIBERSORT-LM7
1.0
0.5
0.0
-0.5
-1.0
Absolute immune score
B cells
CD8+ T cells
NK
CD4+ T cells
Granulocytes
γδT cells
MoMaDC
CIBERSORT-LM22
0.8
0.6
0.4
0.2
0.0
-0.2
-0.4
Absolute immune score
Naive B cells
Activated CD4+ memory T cells
CD8+ T
Acticated mast cells
M2-Macrophages
M0-Macrophages

Figure S8

Supplementary Tables

Table S1. GEO-GPL570 databases screened for analysis

Author	Year	Country	Sample	Source Accession	PMID
Loi S et al.	2007	Canada	87	GSE6532	17401012
Loi S et al.	2008	Canada	77	GSE9195	18498629
Desmedt C et al.	2010	Canada	120	GSE16446	21422418
Sircoulomb F et al.	2009	France	51	GSE17907	20932292
Li Y et al.	2010	USA	115	GSE19615	20098429
Kao KJ et al.	2011	Taiwan, China	327	GSE20685	21501481
Dedeurwaerder S et al.	2011	Canada	88	GSE20713	21910250
Gendrin C et al.	2010	USA	266	GSE21563	25561709
Sabatier R et al.	2011	France	353	GSE31448	22110708
Clarke C et al.	2013	Ireland	104	GSE42568	23740839
Huang C et al.	2014	Taiwan, China	81	GSE48390	24098497
Fumagalli D et al.	2016	Belgium	94	GSE58984	N/A

Table S2. Harrell's concordance indexes of the IRS, TNM stage, and nomogram in GEO, TCGA and METABRIC cohorts

Cohort	Harrell's concordance indexes (95% CI)		
	IRS	TNM stage	Nomogram
**GEO**	0.63 (0.58–0.68)	0.66 (0.59–0.73)	0.71 (0.64–0.78)
**TCGA**	0.56 (0.49–0.63)	0.73 (0.66–0.80)	0.77 (0.70–0.84)
**METABRIC**	0.52 (0.50–0.54)	0.60 (0.58–0.62)	0.62 (0.59–0.65)

IRS, immunorisk score; HR, hazard ratio; CI, confidence interval.

Table S3. Univariable Cox regression analysis of PTGDS with overall survival in GEO, TCGA and METABRIC cohorts

Cohort	HR	95% CI	*P*-value
GEO	0.67	0.55-0.83	0.0001***
TCGA-BRCA	0.84	0.58-0.98	0.016*
METABRIC	0.93	0.88-0.99	0.027*

HR, hazard ratio; CI, confidence interval; *P < 0.05, **P < 0.01, ***P < 0.001.
